# Atypical KCNQ1/Kv7 channel function in a neonatal diabetes patient: Hypersecretion preceded the failure of pancreatic β-cells

**DOI:** 10.1016/j.isci.2024.110291

**Published:** 2024-06-17

**Authors:** Zhimin Zhou, Maolian Gong, Amit Pande, Anca Margineanu, Ulrike Lisewski, Bettina Purfürst, Han Zhu, Lei Liang, Shiqi Jia, Sebastian Froehler, Chun Zeng, Peter Kühnen, Semik Khodaverdi, Winfried Krill, Torsten Röpke, Wei Chen, Klemens Raile, Maike Sander, Zsuzsanna Izsvák

**Affiliations:** 1 Max Delbrück Center for Molecular Medicine in the Helmholtz Association (MDC), 13125 Berlin, Germany; 2 Experimental and Clinical Research Center (ECRC) of the MDC and Charité Berlin, 13125 Berlin, Germany; 3 Department of Pediatrics, Anhui Provincial Children’s Hospital, Hefei 23000, China; 4 The First Affiliated Hospital of Jinan University, Guangzhou 510000, China; 5 Department of Cellular and Molecular Medicine, University of California San Diego, La Jolla, CA, USA; 6 Department of Pediatrics, University of California San Diego, La Jolla, CA 92037, USA; 7 Department of Pediatrics, Klinikum Hanau, 63450 Hanau, Germany; 8 Department of Biology, Southern University of Science and Technology, Shenzhen 518000, China; 9 Charité, Universitätsmedizin Berlin, Virchow-Klinikum, 13125 Berlin, Germany

**Keywords:** Biological sciences, Endocrinology, Health sciences, Internal medicine, Medical specialty, Medicine, Natural sciences, Physiology

## Abstract

KCNQ1/Kv7, a low-voltage-gated K^+^ channel, regulates cardiac rhythm and glucose homeostasis. While *KCNQ1* mutations are associated with long-QT syndrome and type2 diabetes, its function in human pancreatic cells remains controversial. We identified a homozygous *KCNQ1* mutation (R397W) in an individual with permanent neonatal diabetes melitus (PNDM) without cardiovascular symptoms. To decipher the potential mechanism(s), we introduced the mutation into human embryonic stem cells and generated islet-like organoids (SC-islets) using CRISPR-mediated homology-repair. The mutation did not affect pancreatic differentiation, but affected channel function by increasing spike frequency and Ca^2+^ flux, leading to insulin hypersecretion. With prolonged culturing, the mutant islets decreased their secretion and gradually deteriorated, modeling a diabetic state, which accelerated by high glucose levels. The molecular basis was the downregulated expression of voltage-activated Ca^2+^ channels and oxidative phosphorylation. Our study provides a better understanding of the role of KCNQ1 in regulating insulin secretion and β-cell survival in hereditary diabetes pathology.

## Highlights

• A permanent neonatal diabetes melitus patient carries a homozygous KCNQ1 mutation
• KCNQ1^R397W^ is loss of function and shows atypical electrophysiology in hESC-islets
• Under high glucose, elevated Ca^2+^ flux leads to insulin hypersecretion
• Mutant cells gradually switch phenotype, deteriorate, accelerated by high glucose

## Graphical abstract

**Figure:**
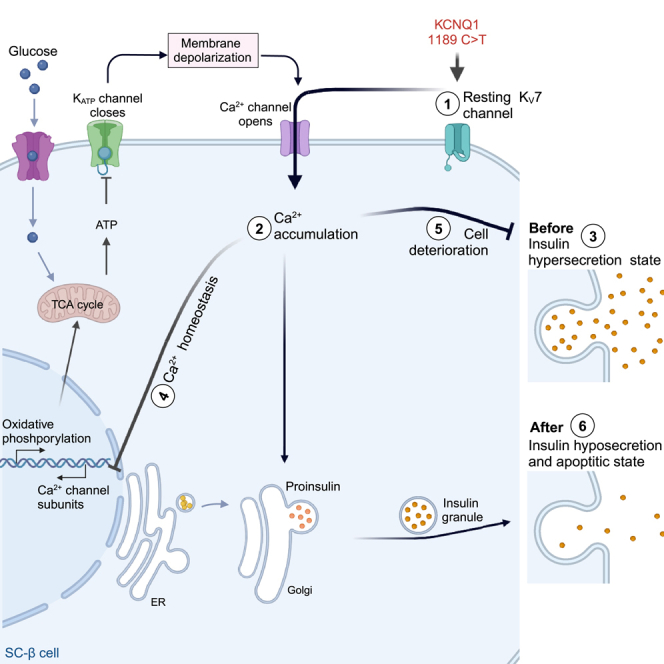

## Teaser

Biological sciences; Endocrinology; Endocrinology; Health sciences; Internal medicine; Medical specialty; Medicine; Natural sciences; Physiology

## Introduction

The low voltage-gated potassium channel of the Q1 subfamily (KCNQ1/Kv7) plays a physiological role in tissues, where it regulates several essential processes, including cardiomyocyte repolarization, vasodilatation, and insulin secretion.^1^^,^^2^^,^^3^^,^^4^ The KCNQ1/K_V_7 channel limits the generation of an action potential in response to depolarization by slowly activating voltage-dependent current and deactivating potassium-selective outward current.^5^^,^^6^^,^^7^^,^^8^ While inherited mutations of KCNQ1 are primarily associated with cardiovascular pathologies (e.g., long QT syndrome 1 (LQT1), familial atrial fibrillation)^4^^,^^9^ and hearing loss,^10^ there are several lines of evidence suggesting that KCNQ1 is also involved in the regulation of insulin secretion.^11^^,^^12^^,^^13^^,^^14^ While both hypersecretory and hyposecretory phenotypes have been reported,^3^^,^^15^^,^^16^ the exact role of the KCNQ1/Kv7 channel in glucose-stimulated insulin secretion remains unclear. Furthermore, the link between KCNQ1-associated cardiac and metabolic syndromes is rather controversial.^17^ The complexity is further increased by the observation that KCNQ1 is part of an epigenetically regulated genomic locus in certain cell types.^18^^,^^19^ The KCNQ1 locus encodes the overlapping regulatory lncRNA (KCNQ1OT1) that controls multiple genes (e.g., *CDKN1C*) in the imprinted genomic region of KCNQ1,^20^ and alteration of *CDKN1C* expression has been reported to affect β-cell mass in humans and mice.^21^^,^^22^ Epigenetic regulation of KCNQ1 expression is important because KCNQ1 is a risk gene that mediates susceptibility to type 2 diabetes.^12^^,^^13^^,^^19^ In contrast, mutations in KCNQ1 have not been associated with neonatal diabetes melitus (NDM). Overall, the exact role of the KCNQ1/Kv7 channel in glucose-stimulated insulin secretion and its association with cardiovascular syndrome(s) needs further clarification.

NDM, a hereditary and monogenic form of diabetes, is usually diagnosed within the first 180 days of life and accounts for only about 0.0012% of all live births.^23^ The best-characterized mutations identified in NDM include *ABCC8, KCNJ11, GCK*, *EIF2AK3* and *INS*.^24^ Patients with NDM exhibit severe β-cell dysfunction, associated with decreased islet cell mass and sometimes even pancreas aplasia.^25^ If insulin is absent during fetal development, the fetus could fail to thrive after birth. Even in milder cases, neonates with NDM are marked for suffering from life-long acute hyperglycemia and life-threatening dehydration.^25^^,^^26^

Here, we report a patient diagnosed with permanent NDM (PNDM), but shows no obvious cardiovascular syndromes. The patient carries a homozygous missense *KCNQ1* mutation (C1189T/KCNQ^R397W^, exon 9), located near the reported translocation (intron 9). Given the careful phenotyping of both homozygous and heterozygous carriers, this variant is of great interest for both cardiac and metabolic phenotypes. The allele frequency is 0.0001875 for this particular C1189T variant (rs199472776) in the GnomAD database (https://gnomad.broadinstitute.org). Interpreted as a disturbed channel function in the cardiomyocytes (LQT1),^4^ the C1189T variant has been also reported as a possible cause of intrauterine fetal death.^27^ Overall, however, there are conflicting interpretations about the contribution of this variant to the cardiac pathology of LQT1.

Motivated by the above challenges, we aimed to decipher the possible contribution of the *KCNQ1* mutation to the pathology, observed in the PNDM patient. To this end, we used CRISPR/Cas9-based genome editing to introduce the KCNQ1 mutation (in hESCs) and subsequently generated islet-like organoids (SC islets^28^). Using our *in vitro* model, we show that the identified mutation (KCNQ1^R397W^) does not affect pancreatic differentiation. Accordingly, our analyses provide no evidence for the epigenetic regulation of this genomic region during pancreatic differentiation. Instead, our data show that the mutation leads to a loss of function of the KCNQ1 channel. The mutant SC islets show atypical extracellular electrophysiology. The impaired channel function leads to a variable, stage-dependent phenotype of insulin secretion. The first hypersecretory SC islets decrease in their insulin secretion and gradually deteriorate, a process that accelerates over time and especially under high glucose conditions, mimicking a diabetic state. While the primary hypersecretory phenotype appears to be relatively mild, the KCNQ^R397W^ mutation induced apoptotic process of pancreatic cells might eventually lead to the observed PNDM phenotype. The detailed functional characterization of KCNQ1 in fully differentiated β-cells allowed us to unravel its role in regulating insulin secretion and β-cell survival.

## Results

### Exome sequencing identifies a homozygous missense KCNQ1^R397W^ mutation in a permanent neonatal diabetes patient

Our patient, born in a consanguineous family at 37 gestational weeks, had intrauterine growth retardation with a birth weight of 1.628 kg and hyperglycemia (293 mg/dL). The QTc value (373ms) was in the normal range, but both the newborn’s insulin and C-peptide levels were below the detection limit, which led to the diagnosis of permanent neonatal diabetes melitus (PNDM). At 10 years of age, our patient’s current condition is stable under regular insulin treatment, and his pancreas is of normal size as determined by echogenicity. Remarkably, both the patient and his family have normal QT intervals and no hearing problems.

**Figure 1:**
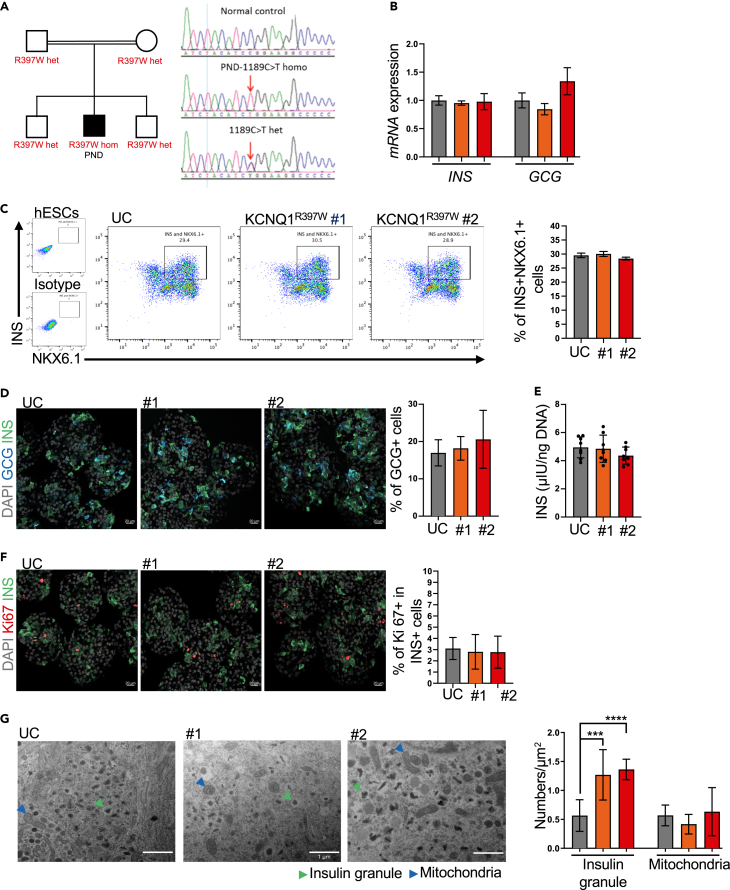
The KCNQ1^R397W^ mutation has no effect on the differentiation of hESC into β cells (A) Pedigree of the patient’s family. Squares and circles represent males and females, respectively. The patient (marked in black) is diagnosed with permanent neonatal diabetes melitus (PNDM). Other members of his family are healthy. The region of the 1189C>T mutation in KCNQ1 is shown from the control, the homozygous (hom) mutation of the patient, and the heterozygous (het) mutation of the patient’s family. (B) q-PCR analysis insulin (*INS*) and *glucagon* (*GCG*) expression in SC-islet (day 31), *n* = 3. (C) Flow cytometry analysis and quantification of cells using stage-specific markers. β cells (day 31) express NKX6.1 and INS, *n* = 3. (D) Immunostaining and quantification of GCG^+^ cells at SC-islet, *n* = 5. (E) Total insulin content per 1ng DNA of cells between KCNQ1^R397W^ and controls SC-islet, *n* = 8. (F) Immunostaining and quantification of Ki67^+^ in SC-β cells, *n* = 8. Scale bar = 20 μm (E and F). In (B–F), *p* values calculated by Student’s t-test indicates non-significant difference. (G) Quantification of the crystallized insulin granules (green arrows) and mitochondria (red arrows). Scale bar = 1 μm *n* = 12. Data presented as mean ± SD. *p* values calculated by Student’s t-test were ∗∗∗*p* < 0.001, and ∗∗∗∗*p* < 0.0001.

To identify potential mutation(s) contributing to PNDM, the patient’s genomic DNA was first analyzed by Sanger sequencing. This analysis excluded the known causative genes of monogenic diabetes (*ABCC8, KCNJ11, INS, GCK, PDX1, SLC2A2, RFX6, EIF2AK3,* and *SLC19A2*). Whole-exome sequencing identified 35 homozygous variants, which were further analyzed using our established pipeline.^29^ This pipeline uses inheritance and functional predictions and provided a short list of potentially disease-causing genes (e.g., *MIA3, KCNQ1, NAXD/CARKD* and *MYO1F*). Compared to 200 normal controls,^30^ only MYO1F and KCNQ1 variants were found in patients. However, as *MYO1F* is not expressed at significant levels in human islets,^19^ we focused our follow-up studies on the KCNQ1 variant to decipher its potential contribution to PNDM. Finally, Sanger sequencing confirmed the nucleotide change C1189T in exon 9 of the *KCNQ1* gene, resulting in a missense mutation (R397W). The PNDM patient is homozygous for this mutation, whereas his parents and brothers are heterozygous and healthy (Figure 1A).

### The KCNQ1^R397W^ mutation has no effect on the differentiation of SC into β cells

To mimic the effects of the C1189T mutation in the patient’s β-cell, we used the CRISPR/Cas9-dependent homology-directed genome editing system^31^ to introduce the homozygous point mutation in hESCs_H1. To investigate potential off-targets, we sequenced the seven top loci predicted by gRNA design tool and found no off-target events at these loci. To mitigate off-target effects not captured by the computational prediction, we generated two *KCNQ1* mutant clones (KCNQ1^R397W^) and one unmodified control clone (UC) from the cell library transfected with Cas9 gRNA and ssDNA (Figure S1A). Of note, all colonies exhibited identical hESC morphology and stained positive for SOX2 and OCT4 (Figure S1B), ensuring that the editing process did not affect pluripotency.

Using our protocol,^28^ we successfully generated insulin-producing (INS^+^) SC-islets from the mutant and control clones. Flow cytometry analyses using antibodies against stage-specific markers revealed no differences between the differentiation process of mutant and control clones (Figures S1C–S1F). Expression analysis of glucagon (GCG) and INS genes and quantification of the percentage of GCG^+^ α-cells and INS^+^/NKX6.1^+^ β-cells performed at day 31 also showed no significant differences between the mutant and UC SC-islets (Figures 1B–1D). Consistent with these results, the SC-β cells in the mutants and UC SC-islets had similar insulin content and proliferation rates (Figures 1E and 1F). Overall, these analyses indicate that the mutation has no effect on the generation of SC-β cells.

In β cells, Ca^2+^ plays a decisive role in the formation of secretory granules by structurally organizing insulin into (Zn^2+^)_2_(Ca^2+^)Insulin_6_ crystals.^14^^,^^32^ Secretory granules initially present as non-crystallized (immature) structures, from which morphologically different crystallized (primed) granules are formed. Importantly, the structure of the insulin granules in the SC-β cells closely resembled that reported in an electron microscopy study of human islets^33^ (Figure 1G). The number of mitochondria were also comparable between the mutant and the control (Figure 1G). Furthermore, both the mutant and the control had a similar insulin content, indicating a comparable number of non-crystallised and crystallised insulin granules. However, KCNQ1^R397W^ SC-β cells showed a significantly increased number of crystallised (primed) insulin granules. This observation led us to hypothesise that the increased number of primed insulin granules might promote increased insulin secretion.

### The C1189T mutation abolishes methylated cytosine but has no effect on gene regulation in the imprinted *KCNQ1* locus

The epigenetically regulated imprinted KCNQ1 locus exerts control over the transcription of the lncRNA *KCNQ1OT1* and consequently affects the expression of neighboring genes.^18^^,^^19^^,^^20^ The methylation status of this genomic locus is implicated in the modulation of β-cell mass via the neighboring CDKN1C gene, a cell cycle inhibitor, in both humans and mice.^21^^,^^22^ Therefore, the C1189 mutation may be associated with a change in gene expression in the imprinted genomic region of KCNQ1 and reduced β-cell mass.^19^^,^^21^ In addition, the imprinted KCNQ1 locus is subject to dynamic regulation by chromatin loops involving CCCTC-binding factors (CTCF). Our data-mining strategy revealed that the *KCNQ1*-C1189T variant overlaps with a predicted CTCF motif as detected in GM12892 (human lymphoblastoid) and K562 (human myelogenous leukemia) cells (Figure S2A).^34^^,^^35^

**Figure 2:**
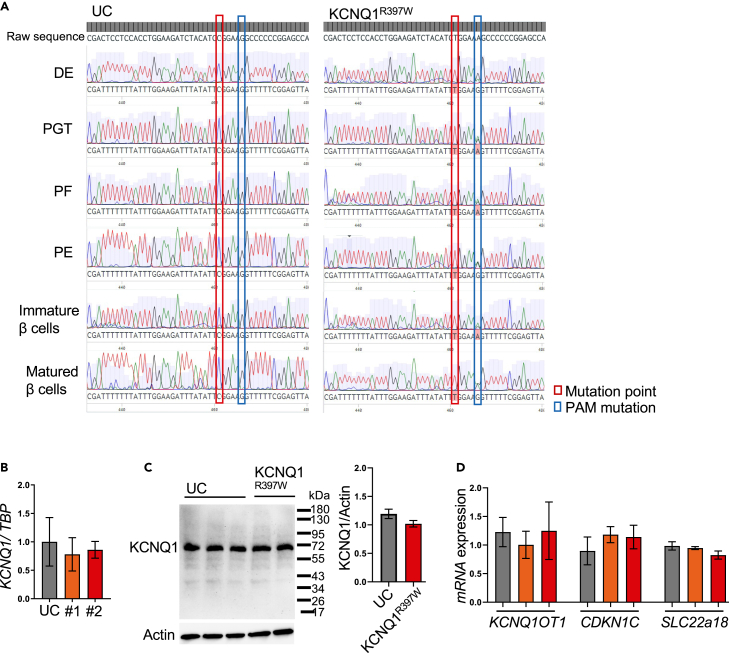
The C1189T mutation abolishes methylated cytosine but has no effect on gene regulation in the imprinted *KCNQ1* locus (A) Cytosine methylation analysis at different stages of differentiation, including definitive endoderm cells (DE), primitive gut tube cells (PGT), posterior foregut (PF), pancreatic endoderm cells (PE), immature β cells and matured β cells. The C1189T mutation is outlined in red, while the PAM mutation is outlined in blue. (B) The *KCNQ1* mRNA level was analyzed by qRT-PCR in SC-islet of UC, and KCNQ1^R397W^ (day 31; #1 and #2), *n* = 3. (C) Western blot analysis and quantification of KCNQ1 expression in SC-islet of control (UC) and KCNQ1^R397W^ (day 31) Data were normalized to ACTIN, *n* = 3. (D) q-PCR analysis of the expressions of *KCNQ1OT1*, *CDKN1C*, and *SLC22a18* in SC-islet of day 31. PHLDA2 was sub-threshold expression levels in all the samples. qPCR data are normalized to housekeeping gene TBP (TATA-Box Binding Protein). Data are presented as mean ± SD. *p* values calculated by Student’s t-test indicates non-significant difference.

To determine the methylation status of the affected cytosine (C1189), we performed a sodium bisulphite conversion analysis. Our analysis revealed that the C1189T mutation (Figure 2A) abolishes DNA methylation. However, C1189 is a continuously methylated cytosine at different stages of differentiation, which makes its regulatory function unlikely (Figure 2A). Consistent with this, there was no evidence of binding of the expected CTCF motif in human islet cells^36^ (Figure S2C). We also analyzed active enhancer signals (H3K27ac) in ChIP-seq data obtained from in vitro-differentiated human β-cells.^37^ This approach revealed β-cell-specific active enhancer signals in intron-11 of KCNQ1, whereas no such signals were detectable in the mutated region (exon 9) at different stages of pancreatic differentiation (Figure S2B).

Accordingly, no mutation-associated changes were detected in the expression studies for KCNQ1 by RT-qPCR and Western blotting (Figures 2B and 2C). Consequently, the overlapping regulatory lncRNA, *KCNQ1OT1* and the other members of the imprinted locus showed either similar or sub-threshold expression levels in all samples (Figure 2D). Overall, the KCNQ1-C1189T variant eliminates DNA methylation but has no affect on the expression of neighboring genes in SC-β cells. This accords with the similar differentiation profiles of wild type and mutant clones and suggests that the PNDM phenotype induced by the mutation is not due to changes in the regulation of gene expression.

### The KCNQ1^R397W^ mutation increases the spike frequency of electrophysiological signals

As an alternative to gene regulation, the C1189T mutation could impair protein function(s). The amino acid of the missense mutation (R397W) is located in a phylogenetically conserved region, and the PolyPhen2 algorithm predicts a potential effect on essential protein function (Figures S3A and S3B). Further analysis with I-TASSER^38^ revealed that the R397W mutation alters the structure of the C- terminal helical region (Helix-A) in KCNQ1 (Figure S3C). Since this region provides interaction surfaces for several interacting partners,^39^^,^^40^ the R397W mutation probably leads to a loss of function of the Kv channel.

SC-β cells, such as native pancreatic β-cells, express a variety of ion channels, including voltage-gated calcium, sodium and potassium channels.^28^^,^^41^ This complexity complicates the isolation of KCNQ1/Kv7 channel currents, which are critical for regulating the threshold and frequency of action potentials during depolarisation.^6^ To accurately study electrophysiological membrane signaling of cells expressing wild-type and mutant KCNQ1 channels without interference from other channel activities, we decided to use CHO-K1 hamster cells for our patch-clamp experiments. This approach ensures precise measurement of KCNQ1/Kv7 channel functions, which is essential for understanding its role in SC-β cell physiology.

**Figure 3:**
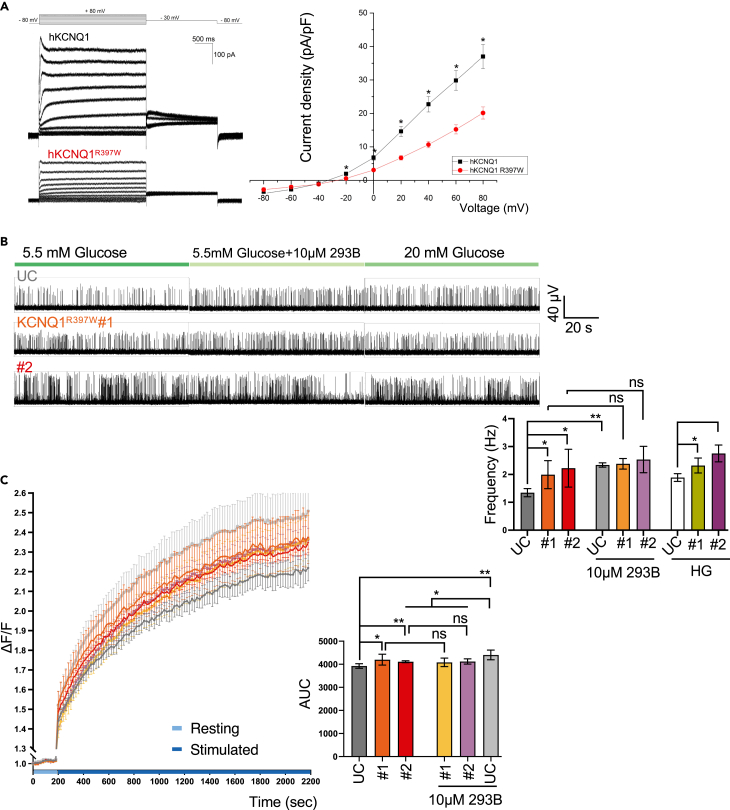
The KCNQ1^R397W^ mutation increases the spike frequency of electrophysiological signals (A) Current traces for human KCNQ1^WT^ (hKCNQ1) and hKCNQ1^R397W^ in transfected KCNQ1-null (The term "KCNQ1-null" refers to the fact that KCNQ1 mRNA levels were undetectable by qPCR.) Chinese Hamster Ovary (CHO-K1) cells (patch clamp). Data presented as mean ± SEM, *p* values calculated by two-way ANOVA. *n* = 3. (B) Recording of extracellular electrophysiology of SC-islet (day 31) induced by 5.5 mM glucose with/without 10 μM Chromanol 293B (293B), and 20 mM glucose (HG). To quantify the frequency, the threshold baseline of the Spike Detector was established based on spikes at a glucose concentration of 3 mM. Spike frequency was quantified after glucose stimulation with/without 10 μM Chromanol 293B (293B), data presented as mean ± SD. *n* = 3. (C) FluxOR labeled thallium assay monitors cations (K^+^, Na^+^ and Ca^2+^) flux. The stimulated moment means the addition of the thallium/potassium stimulus. The data demonstrate the time-dependent fold increase in fluorescence over baseline (ΔF/F) with or without 10 μM 293B, and presented as mean ± SD. The area under of curve (AUC) was quantified upon stimulation, and presented as mean ± SD. *n* = 5. *p* values were calculated using Student’s t-test. ns indicates non-significant difference, ∗*p* < 0.05, ∗∗*p* < 0.01.

To this end, we transfected CHO-K1 cells with expression constructs encoding human KCNQ1^R397W^ or KCNQ1^WT^ (hKCNQ1) and recorded the isolated KCNQ1 current traces (Figure 3A) without co-expression with KCNE1. In contrast to the wild-type profile recorded in cells expressing KCNQ1^WT^, we observed a reduced current density in cells expressing KCNQ1^R397W^ (Figure 3A). Consistent with a previous report^27^ in which KCNQ1^R397W^ was co-expressed with KCNE1 in HEK-293 cells, these observations suggest reduced voltage-dependent activation due to the mutation.

To investigate the relationship between reduced current density and altered subcellular trafficking, we performed immunofluorescence staining and used confocal scanning microscopy and associated software to align, stack, and visualize the confocal images to generate a 3D reconstruction. The results showed that both the KCNQ1^WT^ and KCNQ1^R397W^ proteins were similarly localized to the membrane of transfected CHO-K1 cells and SC-β cells (Figures S3D and S3E), arguing against altered trafficking of the mutant variant to the membrane.

Impaired function of the Kv7 channel in neurons leads to repetitive firing.^42^ To investigate the firing of SC-β cells in response to glucose, we recorded extracellular electrophysiological signals from SC islets exposed to elevated glucose concentrations (normal culture media, 5.5 mM; high glucose conditions, 20 mM). In addition to glucose challenge, we also investigated the effect of Chromanol 293B inhibitor (10μM), which blocks the KCNQ1/Kv7 channel,^43^^,^^44^ and has also been used in patch-clamp studies in cardiomyocytes.^45^

As expected, the UC control showed increased firing in the presence of high glucose or chromanol 293B (Figure 3B). In contrast, KCNQ1^R397W^ SC-islets exhibited increased firing even at low (5.5 mM) glucose concentration (Figure 3B), indicating a dysfunctional KCNQ1/Kv7 channel with accelerated electrophysiological spike frequency. Although Chromanol-293B treatment increased spike frequency in the mutant KCNQ1-expressing cells, the change was not statistically significant, suggesting that the effect of the inhibitor on spike frequency was not additive (Figure 3B).

### Increased Ca^2+^ flux in KCNQ1^R397W^ SC-islets during cultivation with high glucose

**Figure 4:**
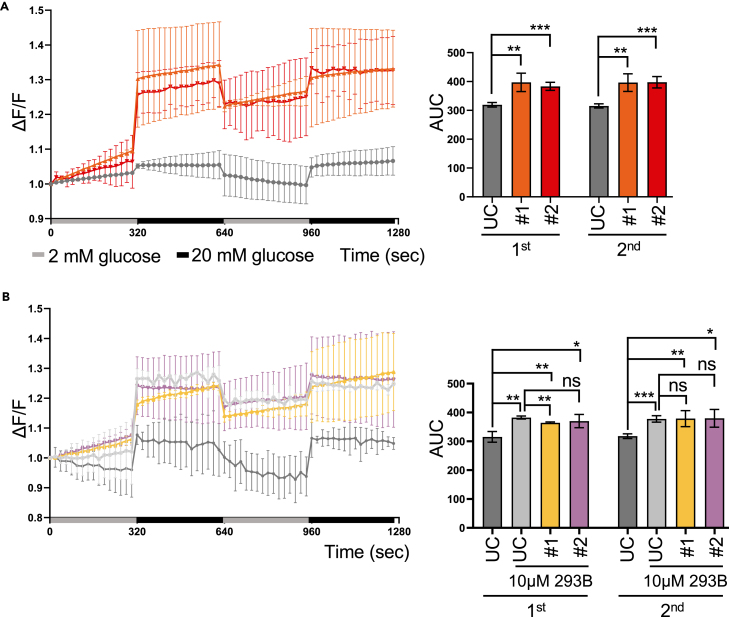
Increased Ca^2+^ flux in KCNQ1^R397W^ SC-islets during cultivation with high glucose (A and B) Dynamic Ca^2+^ flux analysis of SC-islet (day 31) by Fluo-4 a.m. staining. SC-islet were cultured with (B) or without 10 μM 293B (A). Data show the time-dependent fold-increase in fluorescence compared to baseline at 2 mM glucose (ΔF/F), and were presented as mean ± SD. The area under of curve (AUC) was quantified after each stimulation with 20 mM glucose (1^st^ and 2^nd^). *p* values were calculated using Student’s t-test. *n* = 3. n.s indicates a non-significant difference, ∗*p* < 0.05, ∗∗*p* < 0.01, and ∗∗∗*p* < 0.001.

Given the critical role of calcium signaling in synchronizing the periodic change in glucose concentration in β cells,^46^ we aimed to determine how increased electrophysiological spike frequency affects Ca^2+^ levels in KCNQ1^R397W^ SC-β cells. Using Fluo-4a.m.-labelled Ca^2+^ analysis, we subjected SC-islets to high glucose (HG, 20mM) challenges with or without Chromanol-293B at day 31. In response to HG, both SC islets and human islet cells showed increased Ca^2+^ flux (Figures 4A and S3F). Human islets treated with Chromanol-293B, which is known to increase glucose-stimulated insulin secretion (GSIS),^47^ showed an enhanced response (∼1.3-fold) and returned to baseline cytoplasmic Ca^2+^ levels between HG exposures (Figure S3F). Compared to UC, mutant SC islets showed a relatively robust response (∼1.2-fold), but their intensity did not return to baseline during their "recovery" stage (at low glucose, 2 mM) (Figure 4A). Exposure of UC and mutant SC islets to chromanol 293B inhibitor (Figure 4B) resulted in a similar intensity profile, with UC islets not fully recovering from the challenge, indicating a slow response in synchronising the periodic change in glucose concentration. These observations suggest that β-cells with impaired KCNQ1 channels can accumulate abnormally elevated cytosolic Ca^2+^ levels under high glucose concentrations. Notably, isradipine, an L-type Ca^2+^ channel blocker,^48^ could counteract this accumulation (Figure S3G). The similar level of Ca^2+^ influx between UC-SC islets treated with Chromanol-293B and mutant SC islets under high glucose conditions suggests that the effect of the KCNQ1^R397W^ mutation on channel activity may not be directly additive to the effects of Chromanol-293B.

### KCNQ1^R397W^ SC-islets have a variable phenotype of insulin secretion depending on their maturation stages

**Figure 5:**
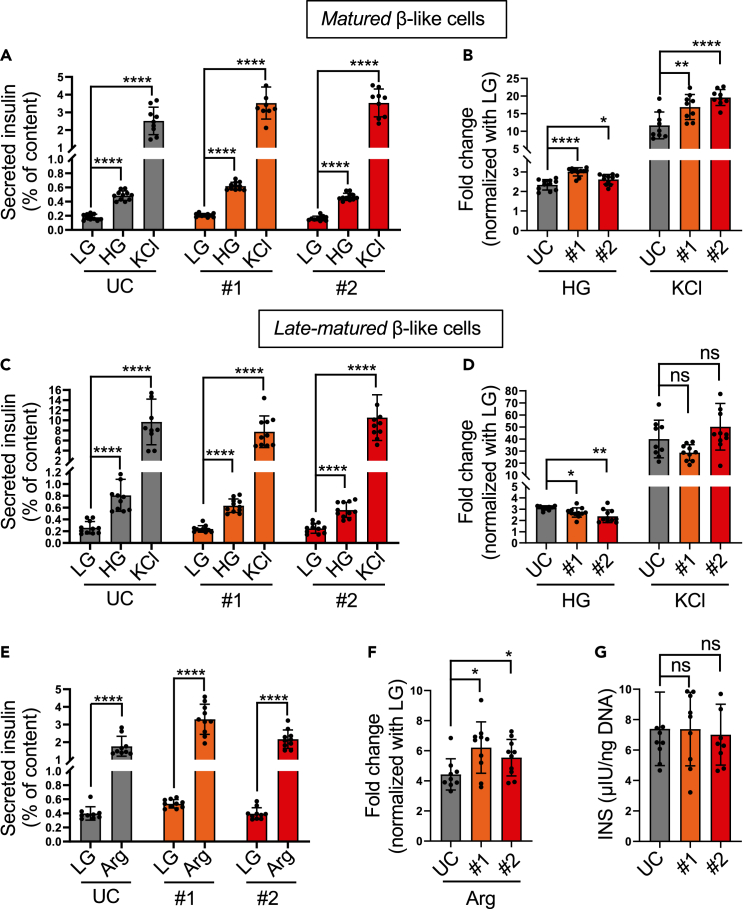
KCNQ1^R397W^ SC-islets have a variable phenotype of insulin secretion depending on their maturation stages (A–D) Insulin secretion of UC and KCNQ1^R397W^ (#1 and #2) SC-islet (% of total insulin content), and fold-change in insulin secretion between 2 mM glucose (LG) and 16.8 mM glucose (HG) or 30 mM KCl (KCl) stimulation at day 31 (A and B) and day 40 (C and D). (E) Insulin secretion of day 40 UC and KCNQ1^R397W^ (#1 and #2) SC-islet (% of total insulin content) with 10 mM Arginine (Arg) stimulation. (F) Fold-change of insulin secretion between LG and 10 mM Arg stimulation in day 40 UC and KCNQ1^R397W^ SC-islet. (G) Total insulin content per 1ng DNA of INS^+^ cells between KCNQ1^R397W^ and UC day 40 SC-islet. Data are presented as mean ± SD. *n* = 10. *p* values calculated by Student’s t-test. n.s indicates a non-significant difference, ∗*p* < 0.05, ∗∗*p* < 0.01, and ∗∗∗∗*p* < 0.0001.

To investigate the effects of the mutation on insulin secretion, we examined glucose-stimulated insulin secretion (GSIS) or KCl depolarization (KSIS) at different time points during *in vitro* differentiation: *maturing* (day 28), *matured* (day 31) and *late matured* (day 40) stages. Compared to control SC-islets, KCNQ1^R397W^ SC-islets responded to high glucose or KCl challenges with increased insulin secretion at day 28 and day 31 (Figures 5A, 5B, S4A, and S4B), aligning with symptoms of postprandial hyperinsulinaemia in a study of 14 patients whose KCNQ1 function was lost.^17^ However, KCNQ1^R397W^ SC-islets no longer responded to the challenges with increased insulin secretion at day 40 (Figures 5C and 5D). The insulin secretion was either comparable to that of the control SC-islets at day 40 or even lower in the KCl depolarization and glucose stimulated assays, respectively. Overall, the mutant SC-islets exhibited a variable, stage-dependent phenotype of insulin secretion, showing unusually high insulin secretion at the *matured*/*maturing* stages and a reversal of this trend after prolonged cultivation (*late matured*).

Considering that intracellular Ca^2+^ can modulate GSIS in β-cells,^49^^,^^50^ we wondered whether insulin secretion could be still stimulated from an intracellular depot at day 40. To this end, we treated day 40 SC-islets with arginine (Arg), which is known to increase Ca^2+^ release from the endoplasmic reticulum (ER) and depolarize β-cells.^51^^,^^52^^,^^53^ Unlike KCl-induced depolarization at day 40 (Figures 5C and 5D), KCNQ1^R397W^ SC-islets responded to Arg with increased in insulin secretion (Figures 5E and 5F). Since their insulin content remained unchanged (Figure 5G), the observed response suggests that Ca^2+^ release from the intracellular depot may still enhance the process of insulin secretion from day-40 KCNQ1^R397W^ SC-islets.

### Prolonged cultivation of KCNQ1^R397W^ SC-β cells leads to the reduced expression of genes associated with oxidative phosphorylation and voltage-activated Ca^2+^ channels

**Figure 6:**
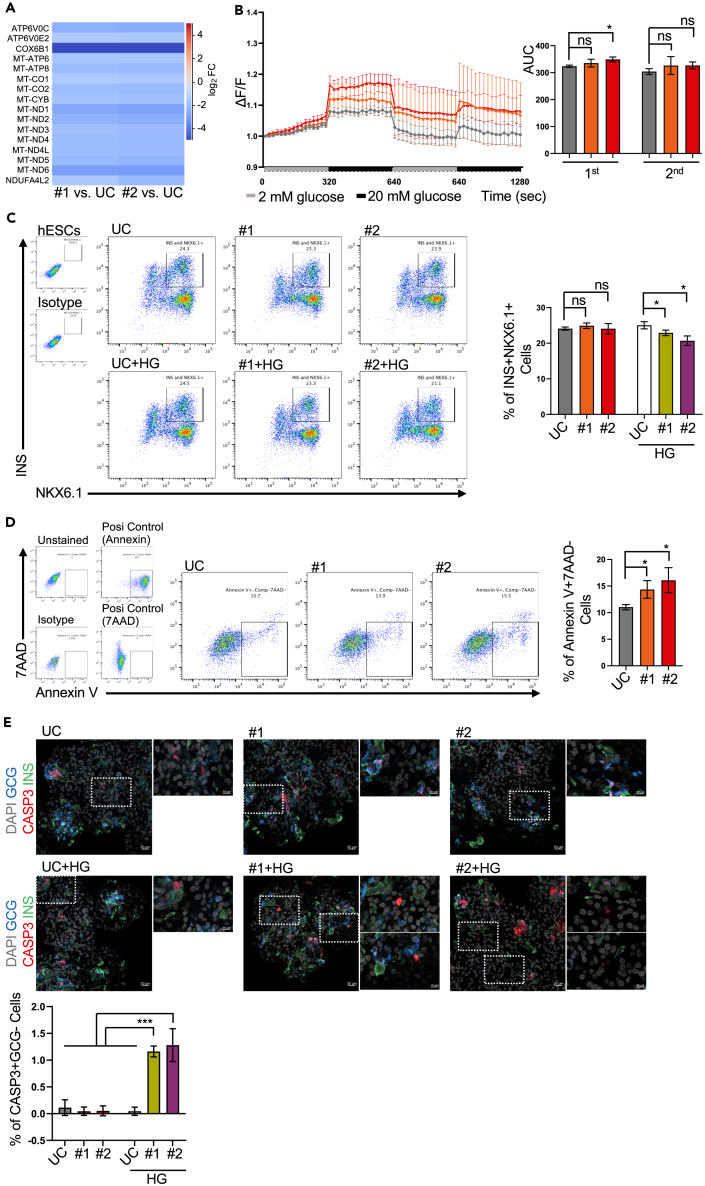
Chronic exposure to high glucose promotes the irreversible deterioration of KCNQ1^R397W^ SC-β cells (A) Heatmap showing the differentially expressed genes (DEGs) in the Gene Ontology category of *oxidative phosphorylation* revealed by RNAseq analysis (three replicates per sample). (B) Dynamic Ca^2+^ flux analysis of day 40 SC-islet by Fluo-4 a.m. staining. The data demonstrate the time-dependent fold increase in fluorescence over baseline in the situation of 2 mM glucose (ΔF/F), and presented as mean ± SD. The area under of curve (AUC) was quantified upon glucose stimulation (20mM). Data presented as mean ± SD and *p* values were calculated using Student’s t-test. *n* = 3. (C) Flow cytometry analysis and quantification of INS in NKX6.1-expressing SC-β cells. SC-islet were cultured in normal S7 media (controls) or in S7 media supplemented with 20 mM glucose (high glucose, HG) for 9 days (from day 32 to day 40), and subjected to flow cytometry (Student’s t-test). *n* = 3. (D) Flow cytometry analysis and quantification of the early apoptotic (Annexin V^+^/7AAD) cells in day 40 SC-islet. As a positive control, cells were treated with 200 μM H_2_O_2_ for 6 h. The data are presented as mean ± SD (Student’s t-test). (E) Day 40 SC-islet were cultured in normal media or supplemented with 20 mM glucose (+HG, high glucose). Immunoassays for DAPI (gray), glucagon (GCG^+^, blue), INS^+^ (green), and cleaved_caspase 3 (CASP3, red). Note that the cleaved_CASP3 Ab can unspecifically bind ɑ/GCG^+^ cells (dual GCG^+^/INS^+^ signals). The original scale bar = 20 μm, and the scale bar of amplified regions is 10 μm. The *p* values were calculated using the Student’s t-test. n.s indicates a non-significant difference, ∗*p* < 0.05, ∗∗*p* < 0.01, and ∗∗∗*p* < 0.001.

To uncover the underlying mechanism(s) for the altered secretion profile of mutant cells following extended cultivation, we performed a transcriptome analysis and determined the differentially expressed genes (DEGs) between UC and KCNQ1^R397W^ SC-islets at day 40. Only DEGs that were common in both mutant colonies compared to UC were considered in the analysis (Figure S5A). The most significant gene ontology (GO) categories (FDR cutoff 0.05) included MAPK signaling (e.g., multiple calcium channel subunits (CACNs)) and oxidative phosphorylation (e.g., COX6B1) (Figures 6A, S5A, and S5B).

Downregulation of several calcium channel subunits (CACNs) (Figure S5B) offers a potential explanation for the UC-like Ca^2+^ flux profiles (Figure 6B) in day 40 KCNQ1^R397W^ SC-islets. Although KCNQ1^R397W^ SC cells possess the required ion channels, they exhibit variations in the expression of ion channel genes (Figure S5B),^28^ which could affect insulin secretion. In addition, the downregulation of the oxidative phosphorylation pathway (Figure 6A) in the day 40 mutant SC-islets suggests a decreased metabolism associated with reduced ATP synthesis in the mitochondria.^54^^,^^55^

Because the accumulation of abnormally elevated cytosolic Ca^2+^ levels under high glucose conditions is a time-dependent process, the KCNQ1^R397W^ mutation could lead to broader cellular effects over time. In addition, mixed channels are present in SC-β cells, so it may be informative to determine the overall activity of cation channels. Furthermore, the mutation and Chromanol-293B differentially affect KCNQ1 channel function, but these potential differences could not be determined from the Ca^2+^ flux experiments (Figure 4). To investigate these aspects, we performed the FluxOR ion channel assay in late maturation (day 40) with or without treatment with chromanol 293B (10μM, from day 32–40). This assay is based on the permeability of different cation channels (K^+^, Na^+^ and Ca^2+^) for thallium^56^^,^^57^ and allows the evaluation of the overall activity of the cation channels upon KCl depolarisation. Our experiments using the FluxOR ion channel assay in the late maturation stage (day 40) showed a higher cation flux profile in KCNQ1^R397W^-hESC islets compared to UC-hESC islets, but the increase was smaller compared to that in UC-hESC islets treated with chromanol-293B (Figure 3C), suggesting a differential effect of the mutation and the inhibitor identified with the assay.

### Chronic exposure to high glucose promotes the irreversible deterioration of KCNQ1^R397W^ SC-β cells

One of the main risk factors for pancreatic β cell loss in diabetic patients is hyperglycemia. We wondered whether prolonged culturing under high glucose (HG, 20 mM) conditions would lead to glucose-induced toxicity in KCNQ1^R397W^ SC-islets. To clarify this, we incubated our day 31 (*matured*) SC-islets for a further nine days (days 40, *late matured*) in stage 7 (S7) media, supplemented or not with high glucose (+HG/Low G). Under +HG conditions, flow cytometry showed a reduced number of SC-β cells (INS^+^/NKX6.1^+^) (Figure 6C), suggesting that KCNQ1^R397W^ SC-β cells are sensitive to high glucose concentrations at day 40. To determine whether induced programmed cell death could be the underlying mechanism, we subjected the cells to Annexin V^+^/7-AAD^-^ assay and immunostaining with antibodies against cleaved_CASP3, INS, and GCG, an apoptosis marker, β-cell marker and an alpha cell-specific marker, respectively. Although, culturing under normal (LG) conditions had no effect on cell numbers (Figures 6C and S5C), we detected a higher number of (Annexin V^+^/7-AAD^-^)-stained KCNQ1^R397W^ cells (Figure 6D), suggesting that the mutant cells exhibit early apoptotic signals. When the day 40 mutant SC-islets were also exposed to +HG conditions, immunostaining revealed the presence of cleaved_CASP3^+^/GCG^−^ cells (Figure 6E), indicating these cells underwent late apoptosis. In our analysis, we noted that cells that stained positive for cleaved_CASP3 frequently did not co-stain with INS, suggesting that these cells may have lost their β-cell identity, possibly as a result of advanced apoptotic processes.

### Impaired KCNQ1/Kv7 channel renders pancreatic β cells sensitive to glucotoxicity

**Figure 7:**
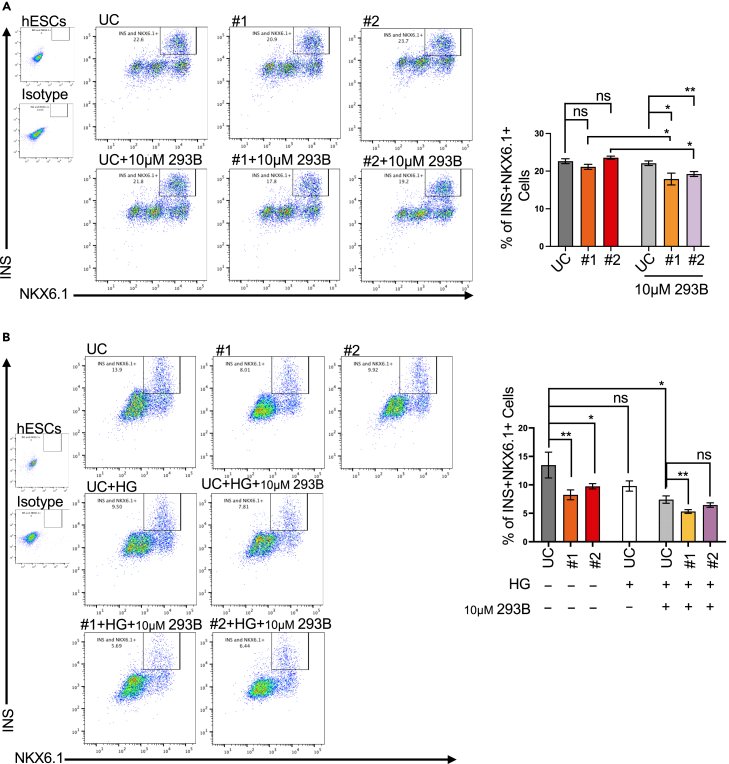
Impaired KCNQ1/Kv7 channel renders pancreatic β cells sensitive to glucotoxicity Flow cytometry analysis and quantification of INS and NKX6.1 expressing SC-β cells. SC-islet were cultured in normal S7 media (controls), in S7 media supplemented with 20 mM glucose (high glucose, HG), or in S7 media supplemented with HG and 10 μM 293B for 2 months (from day 40 to day 100) and subjected to flow cytometry (Student’s t-test). (A) day54 SC-β cells. (B) day100 SC-β cells. Data presented as mean ± SD and *p* values were calculated using Student’s t-test. *n* = 3. n.s indicates a non-significant difference, ∗*p* < 0.05, ∗∗*p* < 0.01.

The above experiments demonstrate the sensitivity of KCNQ1^R397W^ SC-β cells to prolonged cultivation, which can even induce apoptotic cell death in response to high glucose conditions. To find out the fate of KCNQ1-mutated cells, we observed the SC-islets over a further prolonged period. Remarkably, FACS analysis showed a reduced number of KCNQ1^R397W^ SC-β cells after chromanol-293B treatment at day 54 (Figures 7A and S5C), suggesting that the deterioration process could be accelerated in the presence of Chromanol-293B. Without treatment, it took until day 100 for a significant reduction in the number of mutant SC-β cells to occur (Figure 7B). Overall, the impairment of KCNQ1 channel activity, whether by mutation or chemical inhibition, appears to lead to a reduction in cell number, with additive effects on cell mass observed under the combined effects of mutation and inhibition.

In contrast, UC-cell survival remained unaffected even after 2 months of cultivation (day 100) under +HG condition, while the presence of the channel blocker resulted in cell loss in UC SC-islets (Figure 7B), demonstrating that pancreatic β-cells with impaired KCNQ1/Kv7 channel are sensitive to glucotoxicity. Similar to the effects of impaired KCNQ1/Kv7 channel function observed in SC-islets (Figure 7B), immunostaining of KCNQ1^−/−^ mouse pancreatic sections (provided by the Pfeifer laboratory^58^) showed a reduced number of β-cells (Figure S5D).

## Discussion

Here, we report a case of permanent neonatal diabetes melitus (PNDM) in a patient born with no detectable endogenous insulin secretion, carrying a homozygous missense mutation (R397W) in *KCNQ1*. To decipher the aberrant phenotype, we used the CRISPR/Cas9-based genome editing tool in hESCs. Using this approach, we were able to replicate the mutation in pancreatic β-cells and generate a mature SC-islet model, capable of forming secretory insulin granules and responding to glucose-stimulated insulin secretion (GSIS) challenge. Our *in vitro* model provided insights into how the impaired function of the KCNQ1/Kv7 channel leads to a hypersecretory state and eventual loss of KCNQ1^R397W^ SC-β cells. This cascade of events could gradually lead to a hypoinsulinaemic phenotype.

Although the affected KCNQ1 locus is located in an imprinted genomic region, and KCNQ1 is considered a risk gene for type 2 diabetes,^12^^,^^13^^,^^19^ our data show that the C1189T mutation does not alter the epigenetic regulation of gene expression in the imprinted KCNQ1 locus during pancreatic differentiation. Accordingly, the mutation does not affect pancreatic cell differentiation. Therefore, it is unlikely that the phenotype of increased insulin secretion resulting from the mutated KCNQ1 function is due to an acceleration of cell maturation by the mutation.

The missense mutation of R397W impairs channel function. In contrast to the norm, the conserved helical structure (Helix A) is disrupted by the R397W mutation at the C-terminus of the KCNQ1 protein, leading to a loss of function of the Kv channel. KCNQ channels are thought to be controlled by voltage as well as by various interacting protein partners and ligands.^8^^,^^59^^,^^60^^,^^61^ The ligand concentration ranges and interacting regulatory proteins influence channel function and likely have important physiological effects. CaM is an accessory regulatory protein for channel assembly,^39^^,^^62^ and also exerts regulatory control over the KCNQ1 channel gate.^40^ Further functional diversity of the KCNQ1/Kv7 channel results from its association with additional cofactors (e.g., PIP_2_) and protein interaction partners (e.g., KCNE subunits).^8^^,^^60^^,^^61^ Although the mutated residue R397W in KCNQ1 is not directly located in the interaction regions (e.g., PIP_2_, CaM; 225–387, 222–396, 223–396),^61^^,^^63^ the affected helix A (354–397) overlaps, suggesting that a structural change induced by the mutation may affect the functions of KCNQ1 modulated by the interaction partners.

ATP deprivation has been shown to abolish the IK activity of KCNQ1.^64^ The observed downregulation of the oxidative phosphorylation pathway in long-term cultured mutant islets leading to reduced ATP synthesis,^54^^,^^55^ will negatively impact KCNQ1 function and also lead to reduced ATP binding to K_ATP_ channels^14^^,^^65^^,^^66^ and will further strengthen the link between metabolic traits and the altered insulin secretion phenotype of KCNQ1^R397W^-SC islets.

Notably, the R397W mutation has been previously shown to significantly impair the ATP sensitivity of the channel.^67^ Although it has been hypothesized that the R397 residue, together with R380 and K393, likely contributes to the formation of an ATP binding site on the KCNQ1 channel,^8^^,^^67^^,^^68^ the exact role of ATP in KCNQ1 channel function has not yet been fully deciphered. In contrast to ATP, the signaling lipid PIP_2_ is required as a cofactor for the opening of the voltage-sensing pore of KCNQ1.^61^ As an alternative to direct binding, ATP could also modulate the the function of KCNQ1 via the abundance of different ligands, including PIP_2_.

Interestingly, PIP_2_ and ATP have been reported to compete with each other in regulating the opening of the K_ATP_ channel (ATP-sensitive potassium channel). PIP_2_ and ATP antagonistically regulate K_ATP_ channel opening, and mutations (e.g., Q52R and K39R) that increase channel opening by PIP_2_ decrease ATP inhibition and cause neonatal diabetes (NDM).^69^^,^^70^ The potential interplay between ATP and PIP_2_ in the regulation of KCNQ1 function remains to be deciphered.

While the function of KCNQ1 as a voltage-gated potassium channel is primarily characterized for the repolarization phase of the cardiac action potential,^4^ its exact role in human pancreatic β-cells has long been enigmatic. Previous interpretations wrestle with occasional conflicting reports of hypo- and hyper-insulinemic phenotypes.^3^^,^^15^^,^^16^ Our study shows that electrophysiological signals from KCNQ1^R397W^ SC-β cells exhibit increased spike frequency upon glucose stimulation, resulting in elevated Ca^2+^ flux during high glucose challenges. The Ca^2+^ flux is crucial for the crystallization of insulin [(Zn^2+^)_2_(Ca^2+^)Insulin_6_], which forms secretory insulin granules.^14^^,^^32^ This cascade of events triggers enhanced GSIS and KSIS by promoting exocytosis of insulin granules in β-cells. Consistent with our findings, postprandial hyperinsulinaemia and hypoglycaemia symptoms attributable to loss of KCNQ1 function were reported in a study of 14 patients aged over 40 years.^17^

However, the mutant β-cells endeavor to maintain their Ca^2+^ homeostasis in the longer term and their hypersecretion phenotype was attenuated. Decreased expression of high voltage-activated Ca^2+^ channels may provide an explanation for the decline in Ca^2+^ flux to near-normal levels. In the longer term, the mutant cells showed slow deterioration, and chronic exposure to high glucose promoted the irreversible process leading to apoptotic cell death and mimicking a diabetic state.^71^ Our KCNQ1^R397W^-SC islets were suitable for modeling both the hypersecretory and hyposecretory phenotypes and identifying conditions that could provide an explanation for the antagonistic phenotypes.

A similar phenotype, characterized by the switch from hyperinsulinaemia to insulin deficiency, has been reported for other potassium channels.^72^^,^^73^^,^^74^ Dysfunction of the voltage-gated K^+^ (Kv) channel KCNH6 leads to a phenotype of hyper-to hypoinsulinaemia and diabetes in both humans and mice.^73^ In addition, the phenotype of loss/reduced function of the ATP-sensitive potassium channel (K_ATP_) associated with congenital hyperinsulinism (CHI) in humans^74^ is similar to our observations in KCNQ1^R397W^ islets. Indeed, in both the KCNQ1^R397W^ islets and K_ATP_ knockout (K_ATP_-KO) mouse models, cells initially show hypersecretion but transition to a diabetic state over time. Consistent with this, some patients with CHI eventually transition to a diabetic state, suggesting a phenotypic switch.^75^ Furthermore, organoid experiments with the reduced expression of K_ATP_ in MODY3 β-cells have shown that the insulin hypersecretion phenotype precedes pancreatic β-cell failure,^76^ and MODY3 β-cells initially show hypersecretion before eventually manifesting the diabetic phenotype. Collectively, similar to our KCNQ1^R397W^ SC-islets, dysfunction of KCNH6 or K_ATP_ channels leads to the hyperstimulation of insulin secretion in the short term and failure of β-cells in the long term.

In contrast to CHI (and the K_ATP_-KO phenotype), activating or gain-of-function (GOF) mutations in K_ATP_ have been observed in human NDM.^77^ Importantly, however, the underlying mechanisms causing NDM by K_ATP_-GOF and KCNQ1^R397W^ are fundamentally different. Mouse models of human NDM in which K_ATP_-GOF mutations are expressed in β-cells exhibit chronic hypoglycaemia, persistently low [Ca^2+^], and impaired glucose-dependent insulin secretion,^78^^,^^79^ and the loss of β-cell mass results from a shift away from mature β-cell identity toward insulin-negative cells, rather than apoptotic cell death.^80^

Since the dynamic function of β-cells requires an appropriate TGF-β signaling profile, which is only achieved at the final stage of human β-cell differentiation,^81^ it was essential to establish a protocol that could differentiate cells to their mature stage in order to investigate the role of the KCNQ1/Kv7 channel in glucose-stimulated insulin secretion from β-cells.^3^^,^^19^^,^^81^ Similar to human β-cells, our *in vitro* model responded to GSIS and KSIS. Our protocol^28^ worked with hESC_H1, and although patient-derived iPSCs could be an alternative, we were unable to differentiate them to the mature stage of pancreatic differentiation. In our SC islets, following stimulation with high glucose, chaotic Ca^2+^ oscillation of individual SC-β cells could be followed as the sequential fluorescence activation of neighboring cells. Similar to human islets,^82^ SC-islets support the association of endocrine cells and thus the coordination of electrical activity required for insulin secretion.

In agreement with our study, the KO mutation of KCNQ1 (KCNQ1^−/−^) had no effect on pancreatic differentiation.^3^ Nevertheless, the R397W mutation resulted in loss of function and the KO phenotype or that caused by chemical inhibition are slightly different. For example, the KCNQ1^−/−^ SC-islets showed a loss of sensitivity to various secretagogues (KCl, Arg, HG, and so forth), suggesting that the deletion of KCNQ1 may induce adiaphoria,^3^ which we did not observe. While the KO phenotype could represent a genetic compensation for the gene deletion,^83^ the observed differences could be explained by the slightly different differentiation protocols.^3^^,^^28^

The same mutation (R397W) identified in an LQT1 patient^67^ and in a case of intrauterine death^27^ has been shown to reduce the expression of macroscopic hIKs currents. In addition to KCNQ1^R397W^, several KCNQ1 mutations affecting the C-terminal A/B helices of KCNQ1 have been associated with LQT1 syndrome (cardiac arrhythmias),^40^ which predisposes affected individuals to arrhythmias and sudden death. However, although *KCNQ1* may play a role in both cardiac and pancreatic cells, there is no clear link between the cardiovascular and metabolic pathological phenotypes associated with *KCNQ1*. In our example, KCNQ1^R397W^ was identified in a PNDM patient who had no cardiac symptoms.

Our patient and his family show no signs of the cardiovascular phenotype so far (followed for 10 years), suggesting that his disease is caused by defects in insulin production and/or its secretion by β cells. Indeed, our patient has a stable condition on regular insulin treatment. The incomplete clinical penetrance in single families carrying heterozygous *KCNQ1* mutations could partially explain the ambiguity. Furthermore, cardiovascular and metabolic syndromes may appear at different stages in patients. Consistent with this, a study monitoring fourteen LQT1 patients with dominant-negative mutations of *KCNQ1* found that all developed postprandial hyper-insulinemia, but only at ages older than 40 years.^17^ Of note, our transcriptome analysis of KCNQ1^R397W^ SC-islet revealed DEGs enriched in GO categories of cardiac muscle contraction and diabetic cardiomyopathy (Figure S5A). Our ten-years-old patient is also younger than the onset of LQT1 syndrome (the transitional and fatal periods are 12/14 and 32 median age, respectively^84^), so it may simply be too early to draw any conclusions about the cardiovascular aspects of his disease.

In summary, our study sheds light on the complex phenotype associated with the R397W mutation. The mutation does not affect pancreatic differentiation, and the impaired channel function leads to impaired insulin secretion, metabolic derailment and deterioration leading to apoptotic cell death.

### Limitations of the study

Despite the intrauterine growth retardation and loss of pancreatic cells, our patient survived. However, the case of intrauterine death^27^ in particular suggests that the homozygous KCNQ1R397W mutation (possibly in combination with other factors) could even be life-threatening. The rarity of PNDM cases and the difficulty of finding patients with the same mutation remain a challenge. Indeed, further studies and a broader patient cohort are essential to fully clarify the contribution of a disrupted KCNQ1 mutation to hereditary diabetes such as PNDM.

Importantly, our *in vitro* disease model was able to clearly demonstrate a switch from hyperexcitability to a weakened secretory phenotype and an increased susceptibility to toxicity at high glucose load leading to apoptosis. However, when compared to the phenotype observed in patients with permanent neonatal diabetes mellitus (PNDM), our model exhibited a less severe phenotype. Several potential explanations for this discrepancy include the following:

First, the available protocols for the *in vitro* differentiation of human SC islets are largely based on research in mice. However, there are significant developmental differences between humans and mice^85^^,^^86^ that likely impact the dynamics of insulin secretion and cellular excitability. Furthermore, while SC-β cells possess ion channels that are critical for regulating insulin secretion, differences in ion channel composition or regulation persist compared to human islets.^41^

Second, the transition from the hypersecretory to the weakened secretory phenotype of KCNQ1^R397W^ SC islets that eventually leads to a loss of β-cell mass appears to be a slow process. The loss of β-cell identity during the process makes it difficult to accurately estimate the apoptosis rate of SC-β cells and probably leads to an underestimation of the actual number of apoptotic cells. Nevertheless, the progressive decline suggests a stacking effect in which the consequences of the KCNQ1 mutation on β-cell viability become more pronounced over time. This is consistent with the observations reported from the loss-of-function K_ATP_ models.^75^^,^^76^

The above factors may indeed contribute to the milder phenotype of our model compared to that of the patient. However, it is also important to emphasise that despite their limitations, the use of hESC-derived β-cells provides a valuable platform for the study of genetic mutations and their mechanistic effects in a controlled environment. These *in vitro* systems allow the study of specific genetic alterations in a way that is not possible with primary cells, especially when patient samples are unavailable or limited.

## STAR★Methods

### Key resources table

**Table:** 

REAGENT or RESOURCE	SOURCE	IDENTIFIER
**Antibodies**		

PE Mouse anti-Human Sox17	BD Biosciences	Cat#561591
Alexa Fluor® 488 Mouse anti-PDX-1	BD Biosciences	Cat#562274
PE Mouse Anti-Nkx6.1	BD Biosciences	Cat#563023
Alexa Fluor® 647 Mouse Anti-Nkx6.1	BD Biosciences	Cat#563338
Insulin (C27C9) Rabbit mAb (Alexa Fluor® 488 Conjugate)	Cell Signaling Technology	Cat#9016S
Insulin (C27C9) Rabbit mAb (PE Conjugate)	Cell Signaling Technology	Cat#8508S
Anti-Tra-1-60-PE, human	MACS Miltenyi Biotec	Cat#130-122-921
Alexa Fluor® 647 Mouse IgG1 κ Isotype Control	BD Biosciences	Cat#560884
FITC Mouse IgG2a, κ Isotype Control	BD Biosciences	Cat#555573
PE Mouse IgG1, κ Isotype Control	BD Biosciences	Cat#555749
APC Mouse lgG2a, κ Isotype Control	Biolegend	Cat#400219
Rabbit IgG Isotype Control (Alexa Fluor® 488 Conjugate)	Cell Signaling Technology	Cat#4340S
PDX1 Mouse Monoclonal Antibody	Origene	Cat#TA500038
Homeobox protein Nkx-6.1	Developmental Studies	Cat#F55A12
Purified Mouse Anti-Ki-67	BD Biosciences	Cat#550609
Anti-KCNQ1 antibody	ATLAS ANTIBODIES	Cat#R97872
FLEX Polyclonal Guinea Pig Anti-Insulin Ready-to-use	Agilent	Cat#IR002
Insulin (C27C9) Rabbit mAb	Cell signaling Technology	Cat#3014T
Monoclonal Anti-Glucagon antibody	Sigma	Cat#G2654
Sox2 (L1D6A2) Mouse mAb	Cell signaling Technology	Cat#4900S
Oct-4 Antibody	Cell signaling Technology	Cat#2750S
Actin, pan Ab-5	Dianova	Cat#DLN-07273
Alexa Fluor™ 488 Goat Anti-Mouse	Invitroge	Cat#A-11001
Goat anti-Rabbit IgG (H+L) Highly Cross-Adsorbed Secondary Antibody, Alexa Fluor 555	ThermoFisher	Cat#A32732
Goat anti-Guinea Pig lgG (H+L) Highly Cross-Adsorbed Secondary antibody, Alexa Fluor 647	ThermoFisher	Cat#A-21450
HPR-Anti Rabbit lgG (H+L)	Thermo Scientific	Cat#31460
HPR-Anti Mouse lgG (H+L)	Thermo Scientific	Cat#32430

**Bacterial and virus strains**		

*Esherichia coli* DH10β	Invitrogen	Cat#18290015
*Esherichia coli* DH5α	New England Biolab	Cat#C2987H

**Biological samples**		

Human islets	Prodolabs	Cat#HP-20164-01
KCNQ1-/- mouse pancreatic sections	Pfeifer laboratory	https://www.pnas.org/doi/full/10.1073/pnas.041398998

**Chemicals, peptides, and recombinant proteins**		

Activin A	R&D	Cat#338-AC/CF
ALK5 inhibitor II	Enzo Life Sciences	Cat#ALX-270-445
Ascorbic acid	SIGMA	Cat#A4544
CHIR99021	SelleckChem	Cat#S2924
γ-secretase inh. XX	Calbiochem	Cat#565789
Heparin	SIGMA	Cat#H3149
ITS-X	Life technologies	Cat#51500-056
KGF/FGF7	R&D Systems	Cat#251-KG
LDN193189	Stemgent,CA	Cat#04-0074
N-acetylcysteine	SIGMA	Cat#A9165
R428	SelleckChem	Cat#S2841
Retinoic acid	SIGMA	Cat#R2625
ROCK inhibitor	STEMCELL Techn.	Cat#72305
SANT-1	SIGMA	Cat#S4572
T3	SIGMA	Cat#T6397
TPB	Calbiochem	Cat#565740
Trolox	EMD Millipore	Cat#648471
Wnt3A	R&D	Cat#1324-WN/CF
Zinc sulfate	SIGMA	Cat#Z0251
Chromanol293B	Sigma	Cat#C2615
isradipine	Tocris	Cat#2004

**Critical commercial assays**		

High Capacity RNA-to-cDNA kit	Applied Biosystems	Cat#4387406
CloneJET PCR Cloning Kit	Thermo Scientific	Cat#K1231
BCA Protein Assay Kit	Pierce	
QIAGEN plasmid midi kit	Qiagen	Cat#12145
Direct-zol RNA MiniPrep	Zymo research	Cat#R2052
RNA 6000 Nano kit	Agilent	Cat#5067-1511
QIAquick Gel Extraction Kit	Qiagen	Cat#28704
dsDNA Broad Range Kit	DeNovix	Cat#31DSDNA-BR1
SuperSignal™ West Femto Maximum Sensitivity Substrate Kit	Thermo Scientific	Cat#34096
Amersham ECL™ Prime Western Blotting Detection Reagent	Cytiva	Cat#RPN2232
TGX Stain-Free FastCast Acrylamide Kit	BioRad	Cat##1610185
Trans-Blot Turbo transfer system RTA Transfer Kit	BioRad	Cat#1704272
EpiTect Bisulfite Kits	Qiagen	Cat# 59104
Human Insulin ELISA	ALPCO	Cat# 80-INSHU-E01.1
Fixation/Permeabilization Solution Kit	BD Biosciences	Cat#555028
Mix and Go *E. coli* transformation Kit	Zymo Research	Cat#T3001
PE Annexin V apoptosis detection kit	BD Biosciences	Cat#559763
PE Annexin V apoptosis detection kit	BD Biosciences	Cat#559763

**Deposited data**		

RNA-seq raw data	This paper	GSE168245

**Experimental models: cell lines**		

hESCs_H1	WiCell	Cat#WA01
Chinese hamster ovary (CHO-K1) cells	ATCC	Cat# CCL-61

**Oligonucleotides**		

primers, gRNA and ssDNA, see Table S1	LGC	N/A

**Recombinant DNA**		

Px458-GFP	Addgene	Cat#48138
pUC19	Invitrogen	Cat#18290015

**Software and algorithms**		

Fiji	Schindelin et al.^87^	https://imagej.net/software/fiji/
Prism	GraphPad	https://www.graphpad.com/
R	Bell Laboratories	https://www.r-project.org/
EdgeR package	Robinson et al.^88^	https://bioconductor.org/packages/release/bioc/html/edgeR.html

### Resource availability

#### Lead contact

Further information and requests for resources and reagents should be directed to and will be fulfilled by the lead contact, Dr. Zsuzsanna Izsvák (zizsvak@mdc-berlin.de).

#### Materials availability

This study did not generate new unique reagents.

#### Data and code availability

• RNA-seq data are available from the GEO database under accession number GSE168245 and are publicly available as of the date of publication.
• This paper does not report original code.
• Any additional information required to reanalyze the data reported in this paper is available from the lead contact upon request.

### Experimental model and study participant details

#### Patient genetic characterization

A male patient, belonging to the Caucasian race and diagnosed with PNDM was recruited from Charité, Berlin. In the newborn, both insulin and C-peptide levels fell below the limit of detection. Our decision not to administer an insulin secretagogue was further supported by the patient’s stable glycemic control through exogenous insulin therapy, indicating the effectiveness of the current management strategy. Considering the risks associated with off-label use of an insulin secretagogue in newborns and the absence of clinical indication due to adequate control with ongoing treatment, we concluded that such intervention was unwarranted for our patient at this time.

To identify potential mutations, the genomic DNA of the patient was first analysed by Sanger sequencing for the following previously reported causative genes of NDM (*ABCC8, KCNJ11, INS, GCK, PDX1, SLC2A2, RFX6,* and *SLC19A2*) detected no mutations. The genomic DNA of the patient and his parents were subjected to exome sequencing, using the Agilent SureSelect Human All Exon Kit (Agilent SureSelect v4, 50Mb). The data was analyzed using our established pipeline. After filtering, the four identified, potentially disease-causing variants were further analysed by Sanger sequencing. Genomic DNA of the patient, his parents as well as 200 normal controls (also used in^30^) was tested. The variants in genes *MYO1F* and *KCNQ1* were only confirmed in the patient. The Charité committee approved the study (EA-No EA2/054/11).

#### Cell lines

hESC_H1 cell lines were obtained from WiCell. Chinese hamster ovary (CHO-K1) cells were obtained from ATCC. Cell lines tested negative for mycoplasma contamination.

### Method details

#### Electrophysiology analysis of KCNQ1^R397W^ in CHO-K1 cells

CHO-K1 cells were transiently transfected with human KCNQ1 cDNA or KCNQ1^R397W^ cDNA using SuperFect Transfection Reagent (Qiagen). Electrophysiological data were acquired via a Multiclamp 700B amplifier and a Digidata 1440A acquisition system. To measure transfection efficiency, GFP reporter gene was also included in the expression constructs. Similar transfection efficiencies were chosen in CHO-K1 cells so that the control and mutant conditions could be compared. Data were analyzed using pClamp 10.3 software (Molecular Devices).

#### Generation of KCNQ1^R397W^ mutant hESC cell lines

To quickly identify the engineered cells, we used a CRISPR/Cas9-dependent homology-directed genome editing system co-expressing GFP^31^ (Table S1). The plasmids and ssDNA were transfected into hESC_H1 (WiCell) using the XtremeGENE 9 transfection reagent (Roche). Single colonies were picked from the sorted GFP^+^ cell library and cultured on Matrigel (Corning)-coated 48-well plates. The kit of anti-Tra-1-60-PE and anti-PE MicroBeads were used to minimize spontaneous differentiation in the hESCs colonies (MACS, Miltenyi Biotec). The study was approved by the Robert Koch Institute (AZ: 3.04.02/0147).

#### Generation of SC-β cells

hESCs were developed toward insulin^+^ cells in a suspension-based format on a shaker with our protocols.^28^ The single cells were seeded in mTeSR1 media (Stem Cell Technologies) supplemented with CloneR (Stem Cell Technologies) in 6-well ultra-low attachment plates at 5.5 × 10^6^ cells/well. The plates were cultured on the shaker (Binder) at 100 rpm in a CO_2_ incubator (Binder) for 24 h. Undifferentiated aggregates were cultured in daily differentiation media.

##### S1/S2 basal media

500 mL MCDB131 (Life Technologies) supplemented with 0.75 g NaHCO_3_, 1% GlutaMAX (Life Technologies), 15 mM glucose (Sigma) and 2.5 g fatty acid-free BSA (Proliant Biologicals).

##### S3/S4 basal media

500 mL MCDB131 supplemented with 1.25 g NaHCO_3_, 1% GlutaMAX, 15 mM glucose and 10 g fatty acid-free BSA.

S5/S6 basal media: 500 mL MCDB131 supplemented with 0.75 g NaHCO_3_, 1% GlutaMAX, 20 mM glucose and 10 g fatty acid-free BSA.

Day 0 media: S1/S2 basal media, 100 ng/mL Activin A (R&D Systems), 25 ng/mL mouse Wnt3a (R&D Systems).

Day 1 - Day 2 media: S1/S2 basal media, 100 ng/mL Activin A.

Day 3 - Day 5 media: S1/S2 basal media, 50 ng/mL KGF (R&D Systems), 0.25 mM ascorbic acid (Sigma).

Day 6 - Day 7 media: S3/S4 basal media, 50ng/mL KGF, 0.25 μM SANT-1 (Sigma), 1 μM RA (Sigma), 100 nM LDN-193189 (Stemgent), 200 nM TPB (EMD Millipore), 0.25 mM ascorbic acid, 0.5% ITS-X (ThermoFisher).

The plates were cultured on the shaker at 120 rpm in a CO_2_ incubator from day 8 to day 20.

Day 8 - Day 10 media: S3/S4 basal media, 2ng/mL KGF, 0.25 μM SANT-1, 0.1 μM RA, 200 nM LDN-193189, 100 nM TPB, 0.25 mM ascorbic acid, 0.5% ITS-X.

Day 11 - Day 13 media: S5/S6 basal media, 0.25 μM SANT-1, 0.05 μM RA, 100 nM LDN-193189, 1 μM T3 (Sigma), 10 μM ALK5i II (Enzo Life Sciences), 10 μM ZnSO4 (Sigma), 10 μg/mL heparin (Sigma), 0.25 mM ascorbic acid, 0.5% ITS-X.

Day 14 - Day 20 media: S5/S6 basal media, 100 nM LDN-193189, 1 μM T3, 10 μM ALK5i II, 10 μM ZnSO4, 10 μg/mL heparin, 100nM ɣ-secretase inhibitor XX (Calbiochem), 0.5% ITS-X.

S7 media (day 21-day 100): 500 mL MCDB131 supplemented with 1% GlutaMAX, 10 g fatty acid-free BSA, 5mg heparin, 5mL MEM nonessential amino acids, 84 μg ZnSO4, 500 μL Trace Elements A and 500 μL Trace Elements B. Day 21 aggregates were dissociated to single cells and were seeded in S7 media supplemented with CloneR in 6-well ultra-low attachment plates at 5.5 × 10^6^ cells/well. The plates were cultured on the shaker at 100 rpm in a CO_2_ incubator for 24 h. Day22 aggregates were cultured in standard S7 media without CloneR at 120 rpm.

During the differentiation, quality control was performed by flow cytometry or immunofluorescence analysis with antibodies against stage-specific markers.

#### Flow cytometry analysis

SC-islets were treated with TrypLE (10X, ThermoFisher) to dissociate into single cells. The single cells were re-suspended with cold BD fixation/permeabilization solution following the manufacturer’s instructions (BD Biosciences). Quality control of the differentiation was performed by flow cytometry analysis of stage-specific markers. Corresponding isotypes antibodies were loaded into another suspension aliquot as isotope control. Cells were washed and suspended into 0.2% BSA after aspirating supernatant. The samples were analyzed on a flow cytometer after the compensation setting.

#### Immunofluorescence analysis

SC-islets were fixed in 4% PFA and dehydrated in 30% sucrose (w/v). SC-islets were transferred to the center of flat bottom cryosectioning molds (VWR). The mold was filled with OCT and placed in a dry ice ethanol bath to freeze OCT (VWR). Organoids were embedded in frozen OCT and stored at -80°C. The embedded SC-islets were sectioned by using CRYOSTAT MICROTOM (Thermo Scientific) to a thickness of 10 micrometers. Sectioned slides were washed by DPBS to get rid of OCT. The slide was incubated with respective primary antibody and second antibody solutions after blocking. The nuclei were stained with DAPI (Fisher Scientific). The slides were mounted with VECTASHIELD® Antifade Mounting Medium (Vector Laboratories) and covered with coverslips. The mounted and covered slide was sealed with CoverGripTM Coverslip Sealant (Biotium) and allowed to dry fully before being analyzed on LSM700 inverted fluorescent microscope (Zeiss). The 3D reconstructions were performed using the 3D viewer of Zeiss' Zen software, which is specifically designed for high-resolution image processing and surface profiling of samples. This software allowed us to align, stack and visualise the confocal images to create an accurate 3D model of tiny objects such as cell structures. We used a Z-stack scanning method with Zeiss Zen microscope software, which scans from bottom to top of the cell structure to capture all emitted fluorescence signals. This method ensured comprehensive coverage and detailed visualisation of the cellular structures.

Depending on the antibody combinations, the 405, 488, 555, and 640 nm excitation lasers were used in sequential scans to prevent cross-talk between the detection fluorescence channels. The slides were stored long-term at -80°C.

Following Macros to visualize the positive cells most appropriately for stage-specific markers staining:

run("8-bit");

run("Close-");

run("Dilate");

run("Fill Holes");

run("Adjustable Watershed", "tolerance=0.2");

run("Analyze Particles...", "size=20-Infinity pixel show=Outlines display exclude summarize"); We used “and” (means overlap, under “Image Calculator”) to count the double positive cells.

#### Gene expression analysis (qRT-PCR)

Total RNA was extracted from cells using the Direct-zol RNA MiniPrep Plus kit following the manufacturer’s instructions (Zymo Research) and used for cDNA reverse transcription (Applied Biosystems). Gene expression was assessed on the 7900HT Fast Real-Time PCR System (Applied Biosystems) using the Power SYBR Green PCR Master Mix (Applied Biosystems). Data were normalized to *GAPDH* or *TBP* expression using the ΔΔCt method. The primers of the study are listed in Table S1.

#### KCNQ1 mutation locus methylation analysis

SC-islets were lysed in lysis buffer (100 mM Tris-HCl, 0.5 M EDTA, 10%SDS, 5 M NaCl, 0.05% Protein K) and incubated at 55°C overnight. The lysate was mixed with an equal volume of phenol: chloroform: isoamyl alcohol solution (Roch). The aqueous phase (upper) was mixed with a 10% volume of 3 M sodium acetate (pH 5.2) and a 2-fold volume of -20°C cold 100% ethanol. The mixture was placed at -80°C overnight. The supernatant was carefully removed after DNA was settled down by gravity. The DNA pellet was washed with 70% ethanol. The DNA pellet was allowed to air dry for 15 min before resuspending in Nuclear-free H_2_O (Sigma). Sodium bisulfite conversion of unmethylated cytosines in DNA was based on EpiTect Bisulfite Handbook (Qiagen). We designed primers (Table S1) from MethPrimer to sequence the *KCNQ1* mutation locus.

#### Western blotting

SC-islets were lysed in RIPA buffer (50 mM Tris-HCl pH7.4, 150 mM NaCl, 1 mM EDTA, 1% Triton-100, 1% Na-Deoxycholate and 0.1% SDS). The procedure of protein concentration determination was based on the manual of the Pierce BCA Protein Assay Kit (Pierce). Protein samples were boiled at 95°C for 5 min and were run on a TGX Stain-Free acrylamide gel. The gel was prepared by following the manual of the TGX Stain-Free FastCast Acrylamide Kit (BioRad). The proteins were transferred onto a PVDF membrane (BioRad) following the guidelines of the Trans-Blot Turbo transfer system RTA Transfer Kit (BioRad). The PVDF membrane was blocked and then incubated with KCNQ1 antibodies (ATLAS ANTIBODIES) overnight at 4°C. The PVDF membrane was washed with TBST buffer and incubated with HPR-Anti-rabbit lgG (Thermo Scientific) for 1h at room temperature. For detection of KCNQ1, the PVDF membrane was developed with SuperSignal West Femto Maximum Sensitivity Substrate (Thermo Scientific). The antibodies were removed by the mild stripping buffer (15 g Glycine, 1 g SDS, 10 mL Tween 20, add ddH_2_O to 1 L, pH 2.2). The PVDF membrane was blocked and incubated with Actin antibodies (Dianova) overnight at 4°C. The PVDF membrane was incubated with HPRAnti-mouse lgG (Thermo Scientific) for 1h at room temperature. For detection of Actin, the membrane was developed with ECL reagents (Cytiva). The PVDF membrane was imaged on the ChemiDocTM MP imaging system (BioRad).

#### Insulin secretion analysis

SC-islets were transferred to 6-well ultra-low attachment plates with 5 mL KRB buffer (130 mM NaCl, 5 mM KCl, 1.2 mM CaCl2, 1.2 mM MgCl2,1.2 mM KH2PO4, 20 mM Hepes (pH 7.4), 25 mM NaHCO3, 0.1% BSA) containing 2.75 mM glucose. The plates were incubated for 1 h in a 37°C incubator. 5 organoids/well were transferred to 96-well plates with 10 replicates. Insulin secretion stimulation was measured by sequentially adding 2.75mM glucose KRB buffer, 16.8mM glucose KRB buffer, and 30mM KCl KRB buffer or 10mM arginine KRB buffer. Total insulin was measured by adding the acid ethanol solution. The released and total insulin were measured using the Human Insulin ELISA Kit (ALPCO). The SC-islets were collected into 50 μL/well sonication buffer (10 mM Tirs, 1 mM EDTA, 0.2% Triton-X 100, 0.05% Protein K). The SC-islets were sonicated five cycles (30s ON and 30s OFF) in the Bioruptor Pico Sonication device (diagenode). DNA content was detected by dsDNA Broad Range Assay (DeNovix). Values were normalized to total insulin content or DNA mass.

#### Extracellular electrophysiology analysis

SC-islets were dissociated and re-suspended with cold 5% Matrigel, dotted on recording electrodes. The electrical activity (Spike Detector) was recorded in Neural Spikes mode on MAESTRO Pro (AXION BIOSYSTEMS) exposed to increased glucose concentrations (3 mM glucose to set the threshold baseline; normal culture media, 5.5 mM; high glucose condition, 20 mM) with or without Chromanol-293B (10μM). We exported the data and generated figures of extracellular electrophysiology following code.

import numpy as np

import matplotlib.pyplot as plt

# Read the data from the file

filename = 'input_filename'

data = np.loadtxt(filename, usecols=(0, 2))

# Separate time and voltage data

time = data[:, 0]

voltage = data[:, 1]

# Define the number of subplots and time intervals

num_subplots = 3

time_intervals = [(0, 100), (100, 200), (200, 300)]

# Set the maximum voltage value

max_voltage = 0.06

# Plot the data in each subplot

for i, (start_time, end_time) in enumerate(time_intervals):

 # Get the indices of the data points within the specified time interval

 indices = np.where((time >= start_time) & (time < end_time))

 # Plot the data within the time interval

 axs[i].plot(time[indices], voltage[indices], linestyle='-', marker='o', markersize=1, color='black')

 axs[i].set_title(f'Voltage vs Time ({start_time}s - {end_time}s)')

 axs[i].set_xlabel('Time (s)')

 axs[i].set_ylabel('Voltage')

 # Set the x-axis limits to display the data within the specified time interval

 axs[i].set_xlim(start_time, end_time)

 # Set the y-axis limits to display the data within the specified voltage range

 axs[i].set_ylim(0, max_voltage)

# Adjust the layout for better spacing between subplots

fig.tight_layout()

# Show the plot

plt.show()

The threshold baseline of Spike Detector was set by using 3 mM glucose for further quantification. To avoid detecting signals from cell types other than β-like cells, we focused on the electrode targeted cells of organoid that exhibited increased electrophysiological spike frequency.

#### Additional explanation to the glucose concentrations used in the study

3 mM glucose as an important reference point. This concentration is specifically used to establish a minimum baseline for electrical activity, which serves to filter out random fluctuations or background noise. This ensures that any electrophysiological activity observed at 5 mM and 20 mM glucose can be confidently attributed to genuine cellular responses to glucose stimulation.

5.5 mM glucose: This concentration, which is close to the stimulatory levels in humans (generally between 3.9 and 5.5 mM), is standard in the culture media for the maintenance of SC-β cells. It is important to note that this concentration is not intended to mimic the normoglycaemic state in humans. Here it serves as a starting point from which we can observe both activation and inhibition in response to other glucose concentrations and the channel inhibitor.

By setting 3 mM glucose as the threshold for detecting minimal activity, we can compare the responses at this level with those observed at 5 mM (our low glucose condition) and 20 mM (high glucose condition). Thus, our methodological approach involves comparing between these concentrations and focuses on detecting the changes in SC-β cell activity in a graded manner from a low to a high glucose environment.

#### Cation channel assay and cytoplasmic Ca^2+^ level measurement

Human islet or SC-islets (approximately 20 SC-islets or human islets per well) were plated into a 96-well black plate (ThermoFisher) coated with 1% Matrigel, incubated with FluxOR™ reagent in FluxOR™ cation channel assay or Ca^2+^-sensitive fluorescent probe Fluo4-AM (Life Technologies) in cytoplasmic Ca^2+^ level measurement. Time-series images were acquired using CellR live Imaging System (Olympus). Read the plate every 20 sec, obtaining 200 sec of baseline and 2000 sec stimulated.

To measure cytoplasmic Ca^2+^ level, the wells were washed with prewarmed (37°C) KRB buffer containing 2.5 mM glucose after 24 h. The cell organoids were incubated with 50 μM Ca^2+^-sensitive fluorescent probe Fluo4-AM (Life Technologies) in 2.5 mM glucose KRB buffer for 45 min in a 37°C incubator. The plate was incubated further in a 37°C incubator for 15 min after washing with 2.5 mM glucose KRB buffer. The plate was immediately staged on a Cell R live Imaging System to acquire time-series imaging. Fluo-4 AM was illuminated using an excitation filter 492/18 nm, and its emission was collected between 500-550 nm. Time-series images were recorded at 20-sec intervals for 3-5 organoids within a well through all the conditions within a plate using a 20x dry objective. The progression of glucose challenges and time of the stimulation during imaging was as follows: Imaging started after 5 min incubation in KRB buffer containing 2 mM glucose and ran 16 cycles. The next step was followed by a 5 min incubation in KRB buffer containing 20 mM glucose and ran 16 cycles. Sequential low and high glucose challenges were repeated one more time after washing with low glucose KRB buffer. The imaging of the same organoids was resumed after adding low or high glucose solution by retrieving them at the stored positions. Fluorescence intensity was measured by using Fiji software. The StackReg plugin was applied to anchor the organoid positions throughout the time series. These positions of organoids were added to the ROI manager. The fluorescence intensity of each organoid was measured throughout the time series. Finally, the fluorescence intensities of the same organoid were normalized to its first image. The Ca2+ imaging data had drifted baseline. To avoid the drifted baseline affect the calculation of AUC, we used the ΔF/F of its first image (ΔF/F=1) to normalized AUC. In the Ca^2+^ flux assay, we monitored those organoids that showed increased cytoplasmic Ca^2+^ levels in response to a high glucose concentration (20 mM). This allowed us to move out of signals originating from an organoid that predominantly consists of non-beta-like cells.

#### Electron microscopy

SC-islets (day 31) were fixed in a freshly prepared mixture of 2 % formaldehyde and 2 % glutaraldehyde (Sigma) in 0.1 M phosphate buffer (18.2% 0.1 M KH_2_PO_4_, 81.8% 0.1 M Na_2_HPO_4_ in ddH_2_O) for 1 h at room temperature, followed by fixation at 4°C overnight. Samples were stained with 1% OsO_4_ for 2 h after washing with 0.1 M phosphate buffer. They were dehydrated in a graded ethanol series and propylene oxide and embedded in Poly/Bed^R^ 812 (Polysciences Inc.). Ultrathin sections were contrasted with uranyl acetate and lead citrate. Finally, sections were examined with a Morgagni electron microscope (Thermo Fisher). Digital images were taken with a Morada CCD camera and the iTEM software (EMSIS GmbH, Münster).

#### RNA-seq and data analysis

mRNA quality was checked by using Agilent 2100 Bioanalyzer following the protocol of RNA 6000 Nano Kit. BGI Hongkong prepared the DNA libraries and sequenced the libraries on a DNBseq Eukaryotic-T resequencing.

A 30 million 100 bp paired-end reads were obtained per sample. Discarding low-quality reads, trimming adaptor sequences, and eliminating poor-quality bases were done using FASTX-Toolkit and Trimmomatic. Building index and alignment, the reads were performed using Salmon after discarding outliers with over 30% disagreement. GC content and gene length biases were checked using R package NOISeq to quality control of count data. Mean-variance and PCA were calculated between biological replicates using the tximport package in R. The parameters of lengthscaledTPM were CPM cutoff >2 and sample cutoff 2 between the replicates for the analyzed groups. The RUv package from Bioconductor was used to eliminate batch effects. Therefore, all of the samples were normalized to TMM (weighted trimmed mean of M-values). Gene counts was used for differential expression analysis using the EdgeR package. The gene ontology enrichment was performed using ShinyGo v0.61(KEGG, FDR 0.05). The common genes from categories were selected and made heatmaps using the complex heat map package from Bioconductor.^89^

#### Pro-apoptosis analysis

SC-islets were treated with TrypLE (10X) to dissociate to single cells and then washed with 1 mL 0.2% BSA. The cell pellet was re-suspended with 2 mL S7 media supplemented with CloneR. The cells were incubated in a 1% Matrigel-coated plate for two days in a 37°C incubator. The flat culture cells were treated with TrypLE (10X) to dissociate to single cells and then washed with 1 mL 0.2% BSA. FITC Annexin V antibodies and 7-AAD were loaded into 100 μL cells suspension aliquot to analyze pro-apoptosis following the manufacturer’s instructions (BD Biosciences). FITC isotypes were used as the isotope control. Cells were suspended into 200 μL 1X binding buffer after aspirating supernatant. Cells from a one well of 6-well plate were treated with 200 μM H_2_O_2_ 6 h as a positive apoptosis sample for compensation setting. The samples were analyzed on a flow cytometer after the compensation setting.

### Quantification and statistical analysis

Data were analyzed for normal distribution where applicable. Every sample had independent replicates ≥ 3. All qRT-PCR data were analyzed by the ΔΔCt method. Data were analyzed in GraphPad Prism using unpaired/paired t-tests. Asterisks for statistical significance are displayed as ns: not significant; ∗p < 0.05; ∗∗p < 0.01; ∗∗∗p < 0.001, and ∗∗∗∗p < 0.0001. EdgeR package was used for differential expression analysis in RNAseq.
