# Supplementary material for: Atypical KCNQ1/Kv7 channel function in a neonatal diabetes patient: Hypersecretion preceded the failure of pancreatic β-cells
Source: iScience. 2024 Jun 17;27(7):110291. doi: 10.1016/j.isci.2024.110291 (PMC11269803; doi:10.1016/j.isci.2024.110291)
Supplement: Document S1. Figures S1–S5 and Table S1 [file mmc1.pdf]

iScience, Volume *27*

Supplemental information

Atypical KCNQ1/Kv7 channel function in a neonatal diabetes patient: Hypersecretion preceded the failure of pancreatic β-cells

Zhimin Zhou, Maolian Gong, Amit Pande, Anca Margineanu, Ulrike Lisewski, Bettina Purfürst, Han Zhu, Lei Liang, Shiqi Jia, Sebastian Froehler, Chun Zeng, Peter Kühnen, Semik Khodaverdi, Winfried Krill, Torsten Röpke, Wei Chen, Klemens Raile, Maike Sander, and Zsuzsanna Izsvák

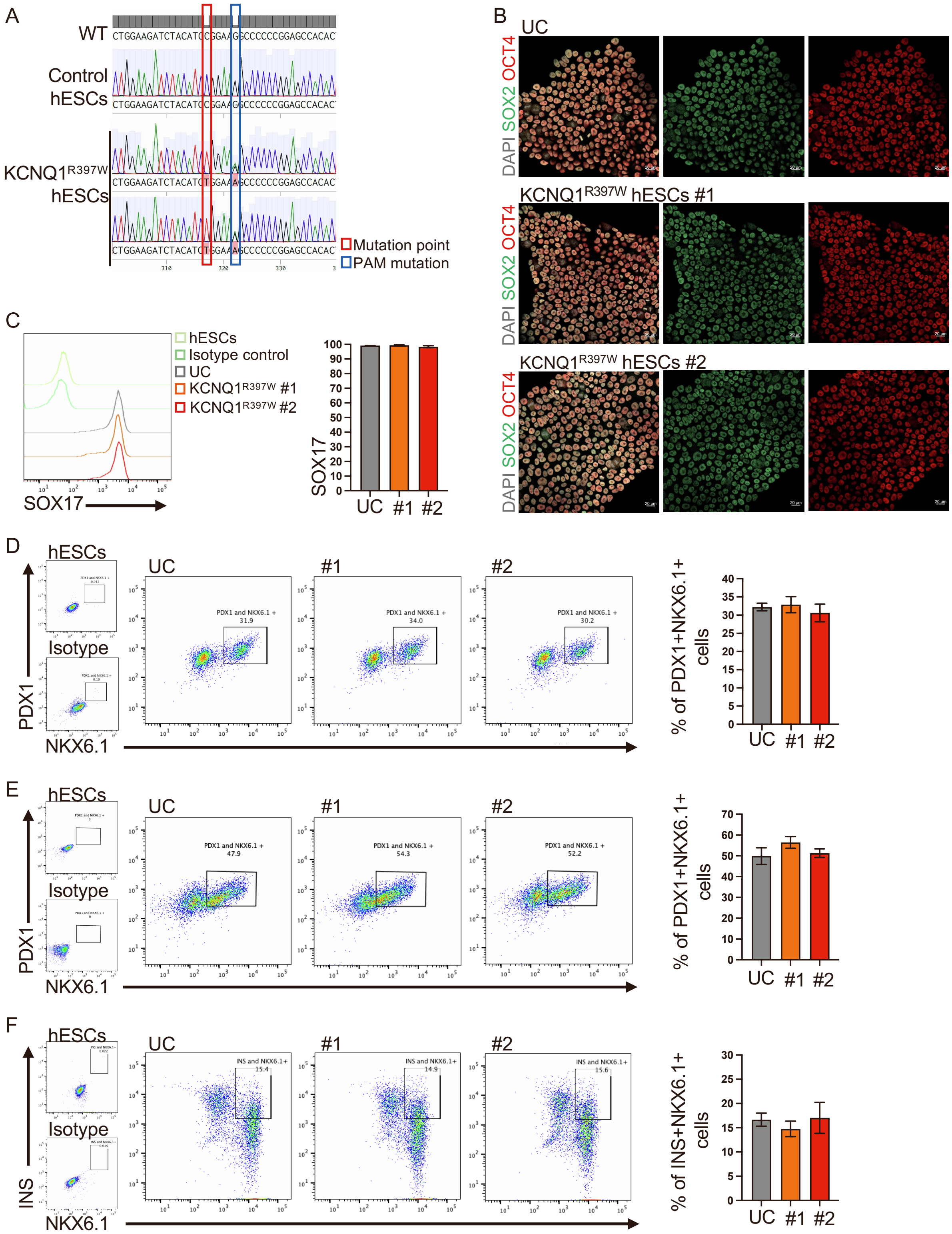

Figure S1. ***In vitro*** **generation of the** ***KCNQ1*** **mutation (1189 C>T) in hESCs, and FACS analysis in different stages of SC-islets differentiation, related to Figure 1.**

A.) Sanger sequencing of CRISPR/Cas9-dependent homology-directed genome edited 1189 C>T mutation (red box), and edited protospacer adjacent motif (PAM) mutation (blue box) in hESCs.

B.) Immunostaining of unmodified control (UC) and KCNQ1^{R397W} hESCs using DAPI (grey) and pluripotency markers SOX2 (green) and OCT4 (red). Scale bar=20 μm.

C-F.) Flow cytometry analysis and quantification of cells using stage-specific markers between UC and KCNQ1^{R397W} (#1 and #2) derived cells. Definitive endoderm cells (day 3) express SOX17 (C). Pancreatic endoderm cells (day 11) express PDX1 and NKX6.1 (D). Pancreatic endocrine precursor

cells (day 14) express PDX1 and NKX6.1 (E). *Immature* β cells (day 21) express NKX6.1 and insulin (INS) (F). Stage-specific cells stained with isotype antibodies and hESCs with stage-specific antibodies are used as negative controls for gating. n=3, data are presented as mean ± SD. p values calculated by Student's t-test indicates non-significant difference.

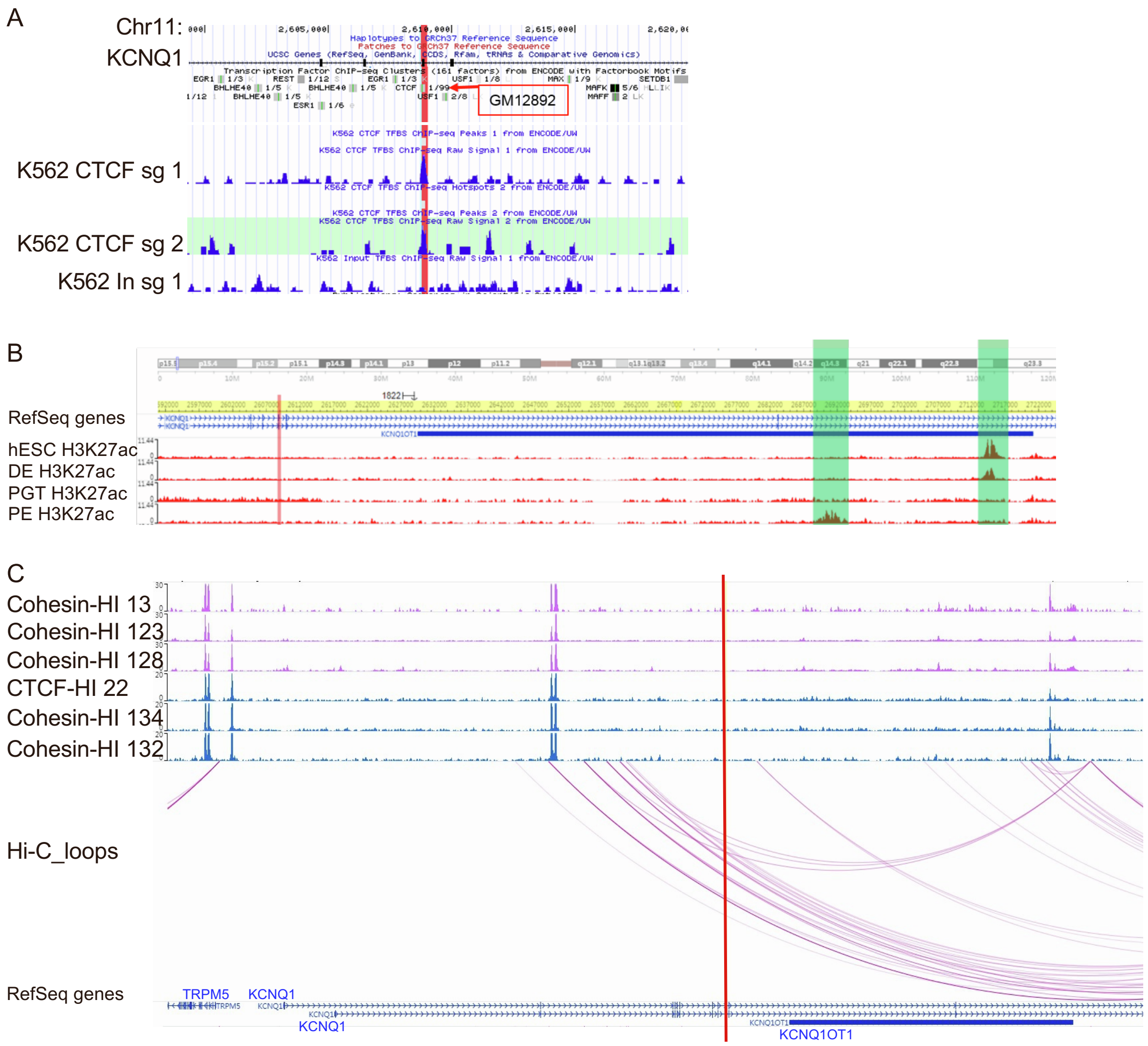

Figure S2. Regulatory genomics of the C1189T mutation in KCNQ1, related to Figure 2.

A.) UCSC Genome Browser shows the CTCF binding signal in KCNQ1 locus. Tracks show signals of CTCF binding (potential CTCF binding motif) in cell lines: GM12892 (human B-lymphocyte, lymphoblastoid and K562 (human myelogenous leukemia).

B.) The dynamic enhancer signal during β-cell differentiation is revealed by H3K27ac CHIP assay[1] in hESCs, definitive endoderm cells (DE), primitive gut tube cells (PGT), and pancreatic endoderm cells (PE). The potential enhancer is highlighted in light green.

C.) Cohesin and CTCF binding (ChIP) signals and the chromatin loops at the KCNQ1 gene and its neighboring genomic region in human islets (Hi-C data[2]).

The position of the C1189T mutation in exon 9 of KCNQ1 is marked by a red bar.

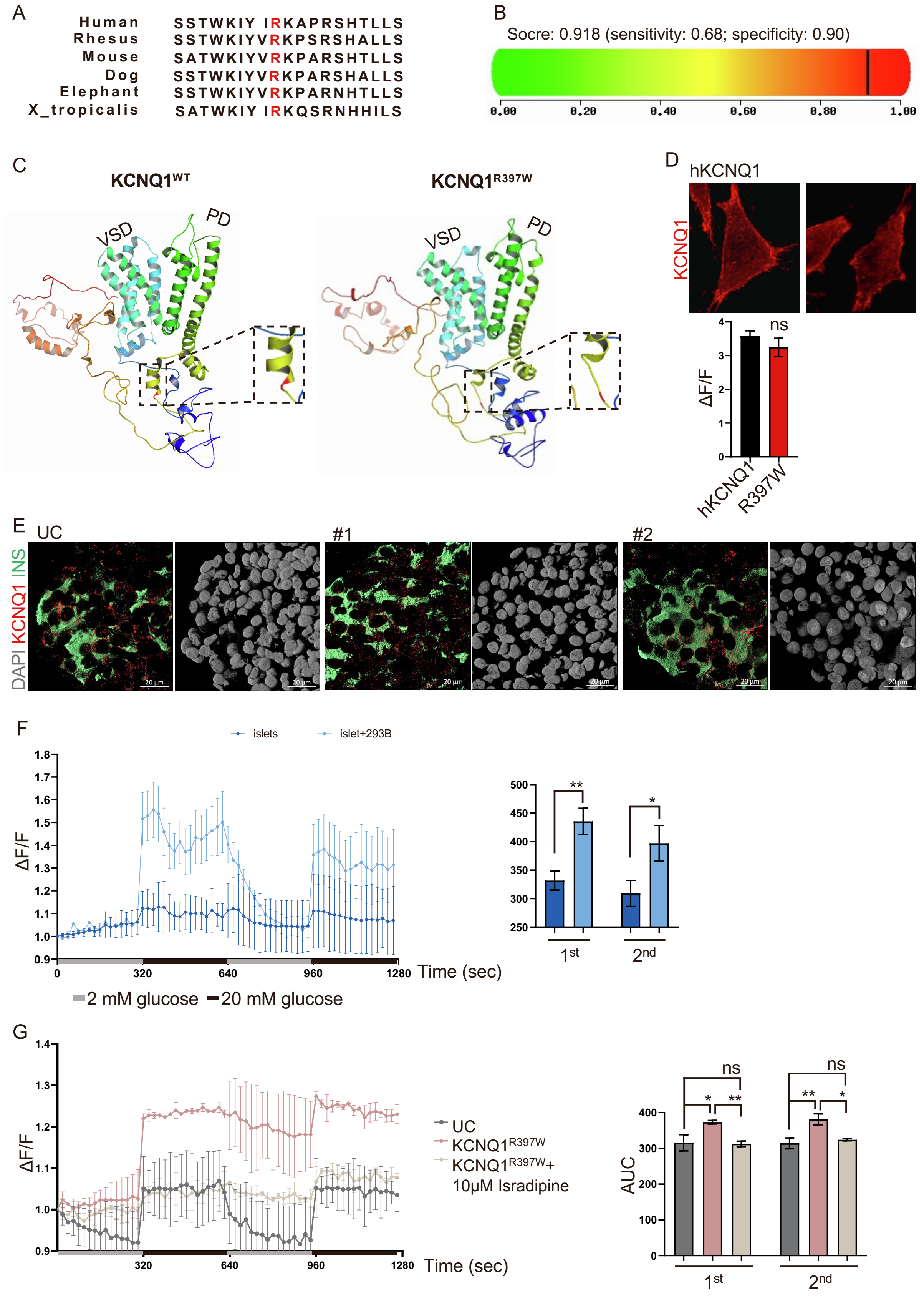

Figure S3. Function prediction of KCNQ1^{R397W} and the dynamic Ca^{2+} flux, related Figure 3 and Figure 4.

A.) Amino acid sequence around the mutation (red) is conserved in KCNQ1.

B.) Polyphen2 prediction for the destructive potential of the R397W mutation in KCNQ1.

C.) 3D structure prediction[3] of human KCNQ1^{WT} and KCNQ1^{R397W}. The figures are generated using the PyMOL viewer. The mutation disrupts the helical structure, resulting in a predicted random coil instead. The mutation locus is highlighted in red.
D.) Immunostaining and fluorescence identity quantification for human KCNQ1^{WT} (hKCNQ1) and hKCNQ1^{R397W} expressed in KCNQ1-null CHO cells (red).
E.) Immunostaining of KCNQ1 (red) and insulin (green) in SC-islet (day 31) showed by 3D confocal scanning. Scale bar=20 µm.
F.) The dynamic Ca^{2+} flux of human islet by Fluo-4 AM staining. Human islets were cultured with or without 10 µM Chromanol 293B (293B). Data are presented as mean ± SD.
G.) The dynamic Ca^{2+} flux of SC-islet by Fluo-4 AM staining. SC-islets were cultured with or without 10 µM isradipine.
Data are presented as mean ± SD. The area under of curve (AUC) is quantified upon two times 20 mM glucose stimulation (1st and 2nd), and p values were calculated using Student's t-test calculates p values. n=3. n.s indicates a non-significant difference, *$p < 0.05$, **$p < 0.01$.

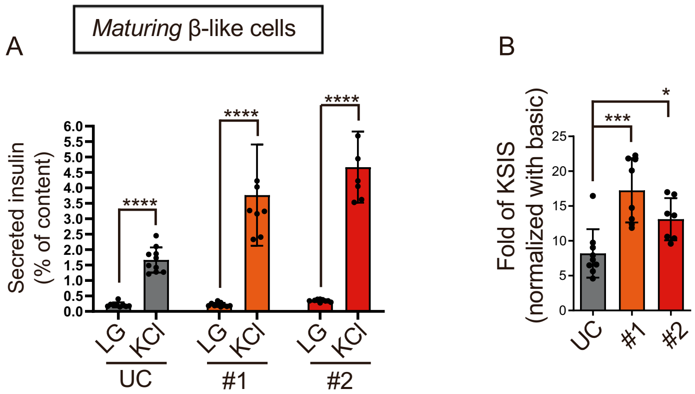

Figure S4. The insulin secretion of maturing SC-islet upon stimulation, related to Figure 5.

A.) The insulin secretion of maturing UC and KCNQ1^{R397W} (#1 and #2) SC-islet (% of total insulin content) with 30 mM KCl stimulation.

B.) Fold-change of insulin secretion in maturing SC-islet (day 28) between 2 mM glucose (LG) and 30 mM KCl stimulation in UC and KCNQ1^{R397W} SC-islet.

Data are presented as mean ± SD. n=9. *$p < 0.05$, ***$p < 0.001$, and ****$p < 0.0001$ (Student's t-test).

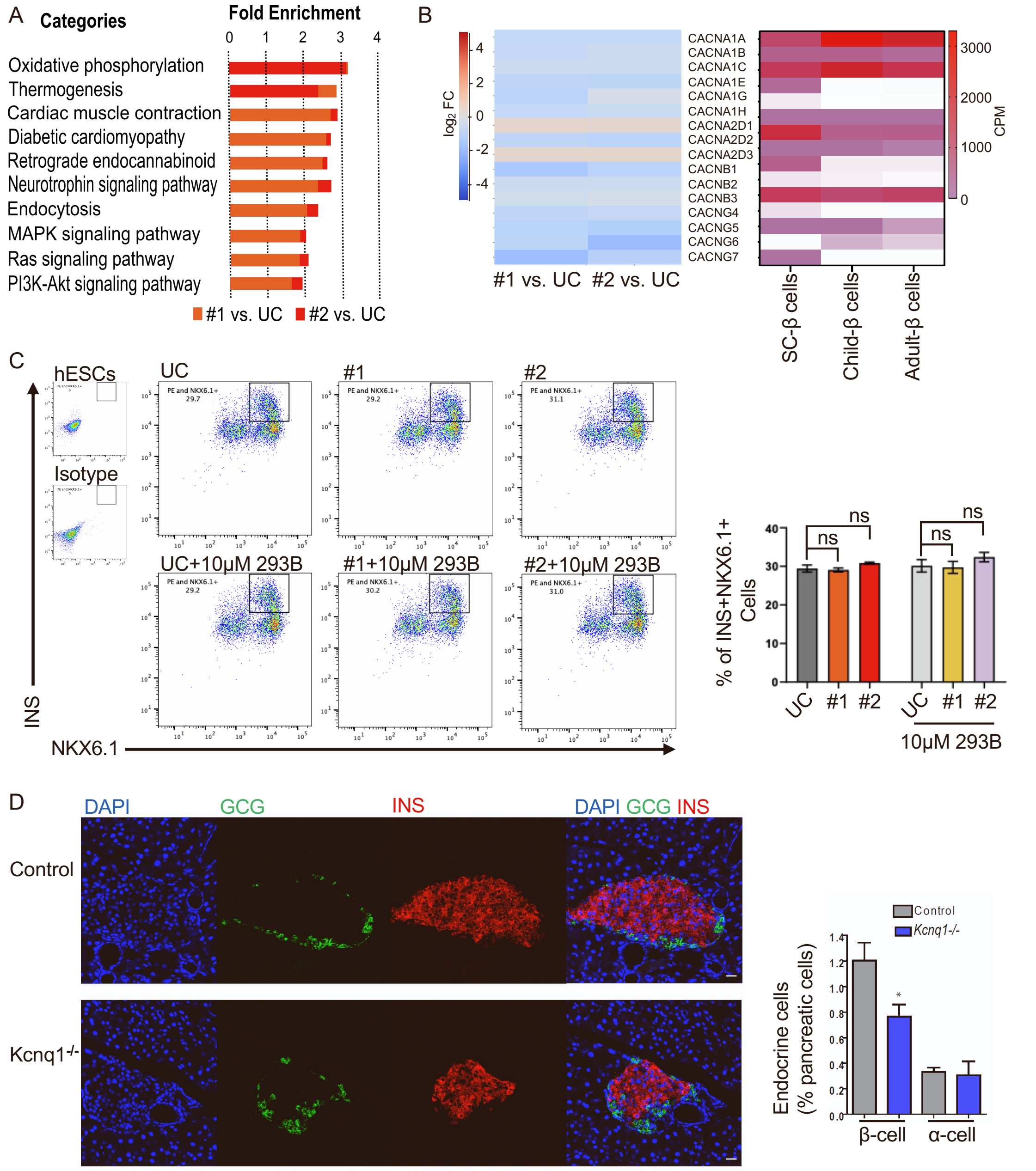

Figure S5. Differentially expressed genes analysis and quantification of β-cell number, related to Figure 6 and Figure 7.

A.) GO analysis of differentially expressed genes (DEGs). The gene ontology (GO) classified only common in both mutated colonies and showed top ten categories (KEGG, FDR cutoff 0.05). Three replicates per sample for RNAseq.

B.) Heat map showing the differentially expressed genes (DEGs) revealed by RNAseq analysis between UC and KCNQ1^{R397W} SC-islet (left panel) and the counts per million (CPM) between SC-, child-, and adult-islet revealed by snRNAseq[4]. The DEGs belong to the GO categories of *Ca^{2+}channel complex*.

C.) Flow cytometry analysis and quantification of INS and NKX6.1 expressing SC-β cells. *Mature* SC-β cells were cultured in normal S7 media (controls) or S7 media supplemented with 10 μM Chromanol 293B for 9 days (from day 32 to day 40) and subjected to flow cytometry.

D.) Immunostaining analysis of KCNQ1-/- mouse pancreas sections (provided by Pfeifer Lab[5]). Immunoassays for DAPI (blue), glucagon (GCG+, green), and INS+ (red). Scale bar=20 μm.
Data are presented as mean ± SD, n=3. n.s indicates a non-significant difference, *p < 0.05 (Student's t-test).

Table S1: The sequence of primers, gRNA and ssDNA， related to STAR Methods.

Name	Usage	Sequence (5'-3')
Exon9	PCR screening	Forward: CCTTCCTGGGCACATACA<br>Reverse: AGCCAACCACGTCCACT
R397W	Mutation generation	Forward:CCACCTGGAAGATCTACATCTGGAAGGCCCCCCGGAGCCACA<br>Reverse:TGTGGCTCCGGGGGGCCTTCCAGATGTAGATCTTCCAGGTGG
Kcnq1_S1	PCR screening	Forward:ACATCAATGGGCGTGGATAG<br>Reverse:GAAGACGGCGAAGTGGTAAA
Kcnq1_S2	PCR screening	Forward: GGCCTGGCCAAGAAGTG<br>Reverse:GGTACACAAAGTACGAGGAGAAG
Kcnq1_S3	PCR screening	Forward: CCGCCAGGAGCTGATAAC<br>Reverse:TTCTTTACCACCACAGACTTCTT
Kcnq1_S4	PCR screening	Forward: TGCTATGCTGCCGAGAAC<br>Reverse:CCTTGTCTTCTACTCGGTTCAG
Kcnq1_S5	PCR screening	Forward: GAAGCCCTCACTGTTCATCT<br>Reverse: TCAGGACCCCTCATCGGG
KCNQ1	PCR amplified the CRISPR-edited genomic region	Forward: CTGGCTCAGGGCTGGTAAAG<br>Reverse: GGAACCTAGCATCGGGTGTA
Bis-KCNQ1	Bisulfite sequencing PCR	Forward:GGAGTTGGGATTATGTTAAGTTTGT<br>Reverse:AAAACCCTCTAAAAAACCTTCTTTC
KCNQ1	Real-Time PCR	Forward: CATCACCCACATCTCACAGC<br>Reverse: GTCCCGCACATCGTAAGG
KCNQ1ot1	Real-Time PCR	Forward: CTTTGCAGCAACCTCCTTGT<br>Reverse: TGGGGTGAGGGATCTGAA
SLC22A18	Real-Time PCR	Forward:CATCTTGCTTACCTACGTGCTG<br>Reverse: CCCAGTTTCCGAGACAGGTA
PHLDA2	Real-Time PCR	Forward:TCCAGCTATGGAAGAAGAAGC<br>Reverse:GTGGTGACGATGGTGAAGTACA
CDKN1C	Real-Time PCR	Forward:AGATCAGCGCCTGAGAAGTCGT<br>Reverse:TCGGGGCTCTTTGGGCTCTAAA
NANOG	Real-Time PCR	Forward:CCAAAGGCAAACAACCCACTT<br>Reverse: CGGGACCTTGTCTTCCTTTTT

OCT4	Real-Time PCR	Forward: CGACCATCTGCCGCTTTG<br>Reverse: GCCGCAGCTTACACATGTTCT
Sox2	Real-Time PCR	Forward:ACAGCAAATGACAGCTGCAAA<br>Reverse: TCGGCATCGCGGTTTTT
Insulin	Real-Time PCR	Forward: AGCCTTTGTGAACCACACC<br>Reverse: GCTGGTAGAGGGAGCAGATG
FGFRL1	Real-Time PCR	Forward: CCTGAGCGTCAACTACACC<br>Reverse: CTCATCTTGGAGGGCTGTG
FGFR1	Real-Time PCR	Forward:AACCTGACCACAGAATTGGAGGCT<br>Reverse:ATGCTGCCGTACTCATTCTCCACA
FGFR3	Real-Time PCR	Forward: AGGTGAATGGCAGCAAGGT<br>Reverse: CTAGCTCCTTGTCGGTGGTG
PDX1	Real-Time PCR	Forward:AAGTCTACCAAAGCTCACGCG<br>Reverse: GTAGGCGCCGCCTGC
GLUT1	Real-Time PCR	Forward: TCCCTGCAGTTTGGCTACA<br>Reverse: GTGGACCCATGTCTGGTTGT
GLUT2	Real-Time PCR	Forward: CACCAATTCCAGCTACCGAC<br>Reverse: CCGTCTGAAAAATGCTGGTT
Glucagon	Real-Time PCR	Forward:GAGGAAGGCGAGATTTCCCAG<br>Reverse: GAACCATCAGCATGTCTGCG
HNF4α	Real-Time PCR	Forward: CAAACACTACGGTGCCTCG<br>Reverse: GTCTTTGTCCACCACGCACT
KCNQ1g-1	sgRNA+BpiI sticky end	Forward:CACCGCCCGACCTCAGACCGCATG<br>G<br>Reverse:AAACCCATGCGGTCTGAGGTCGGG<br>C
KCNQ1g-2	sgRNA+BpiI sticky end	Forward:CACCGCCACCTGGAAGATCTACATC<br>Reverse:AAACGATGTAGATCTTCCAGGTGGC
KCNQ1t-1	ssDNA	CTGGGTGACAGCAGAGTGTGGCTCCGGGG<br>GGCCTTCCGGATGTAGATCTTCCAGGTGGA<br>GGAGTCGGGGTTCTCGGCAGCATAGCATCT<br>CCATGCGGTCTGAGGTCGGGCAGGGGGAC<br>AGGCTGTCAC
KCNQ1t-2	ssDNA	GCCCTGGTGGCAGGTGGGCTACTCACCACC<br>ACAGACTTCTTGGGTTTGGGGCTGGGTGAC<br>AGCAGAGTGTGGCTCCGGGGGGCCTTTCG<br>GATGTAGATCTTCCAGGTGGAGGAGTCGGG<br>GTTCTCGGC

Uncategorized References:

S1. Xie, R. *et al. Cell Stem Cell* 12, 224-237, (2013), 'doi':10.1016/j.stem.2012.11.023 Available at: http://www.ncbi.nlm.nih.gov/pubmed/23318056.

S2. Miguel-Escalada, I. *et al. Nat Genet* 51, 1137-1148, (2019), ‘doi’:10.1038/s41588-019-0457-0 Available at: http://www.ncbi.nlm.nih.gov/pubmed/31253982.

S3. Zhou, X. *et al. Nat Protoc* 17, 2326-2353, (2022), ‘doi’:10.1038/s41596-022-00728-0 Available at: https://www.ncbi.nlm.nih.gov/pubmed/35931779.

S4. Zhu, H. *et al. Dev Cell* 58, 727-743 e711, (2023), ‘doi’:10.1016/j.devcel.2023.03.011 Available at: https://www.ncbi.nlm.nih.gov/pubmed/37040771.

S5. Casimiro, M. C. *et al. Proc Natl Acad Sci U S A* 98, 2526-2531, (2001), ‘doi’:10.1073/pnas.041398998 Available at: https://www.ncbi.nlm.nih.gov/pubmed/11226272.
